# Supplementary figures and images for: Mapping schistosomiasis risk landscapes and implications for disease control: A case study for low endemic areas in the Middle Paranapanema river basin, São Paulo, Brazil
Source: PLoS Negl Trop Dis. 2024 Nov 4;18(11):e0012582. doi: 10.1371/journal.pntd.0012582 (PMC11563476; doi:10.1371/journal.pntd.0012582)

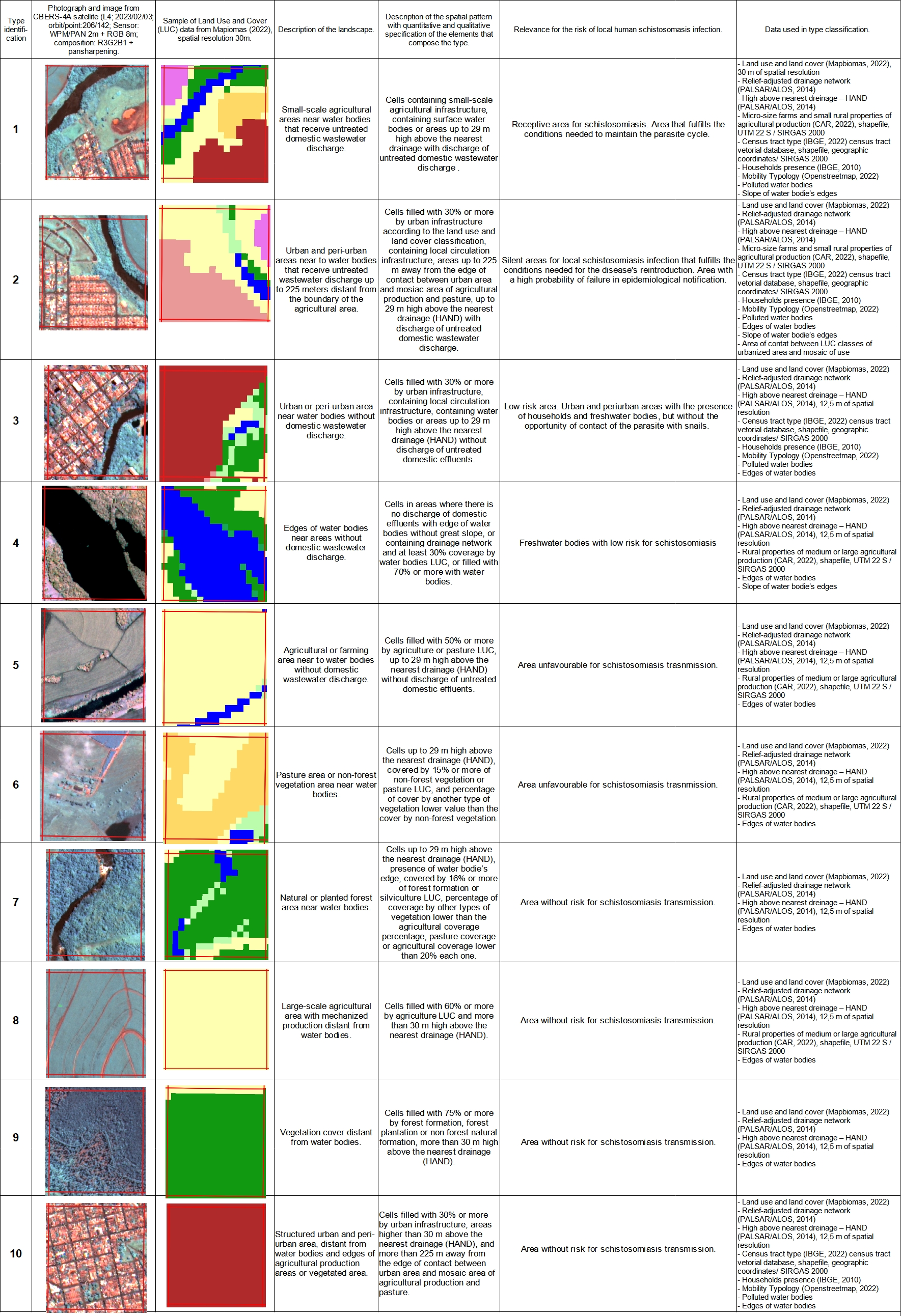

Supplement: S1 Fig — The figure shows 10 types of landscape units associated with schistosomiasis in the MP basin. To describe and characterise these types, we used (i) an image from the CBERS-4A satellite (L4; 2023/02/03; orbit/point:206/142; Sensor WPM/PAN 2m + RGB 8m; composition: R3G2B1 + pansharpening); (ii) a selected sample of land use and land cover data from MapBiomas (2022); (iii) a general description of that landscape associated with the type; (iv) Description of the spatial patterns observed in the landscape with quantitative and qualitative characterisation for the elements that make up that landscape; (v) a description of the relevance of that landscape in the composition of the local risk for schistosomiasis; and (vi) the data used to generate the variables/indexes that took part in the supervised classification process based on a machine learning approach. Satellite image available from http://www.dgi.inpe.br/catalogo/explore. Terms of use available from https://www.gov.br/pt-br/servicos/obter-imagens-de-sensoriamento-remoto-da-terra-geradas-pelo-satelite-cbers-04a. (TIF) [file pntd.0012582.s001.tif]

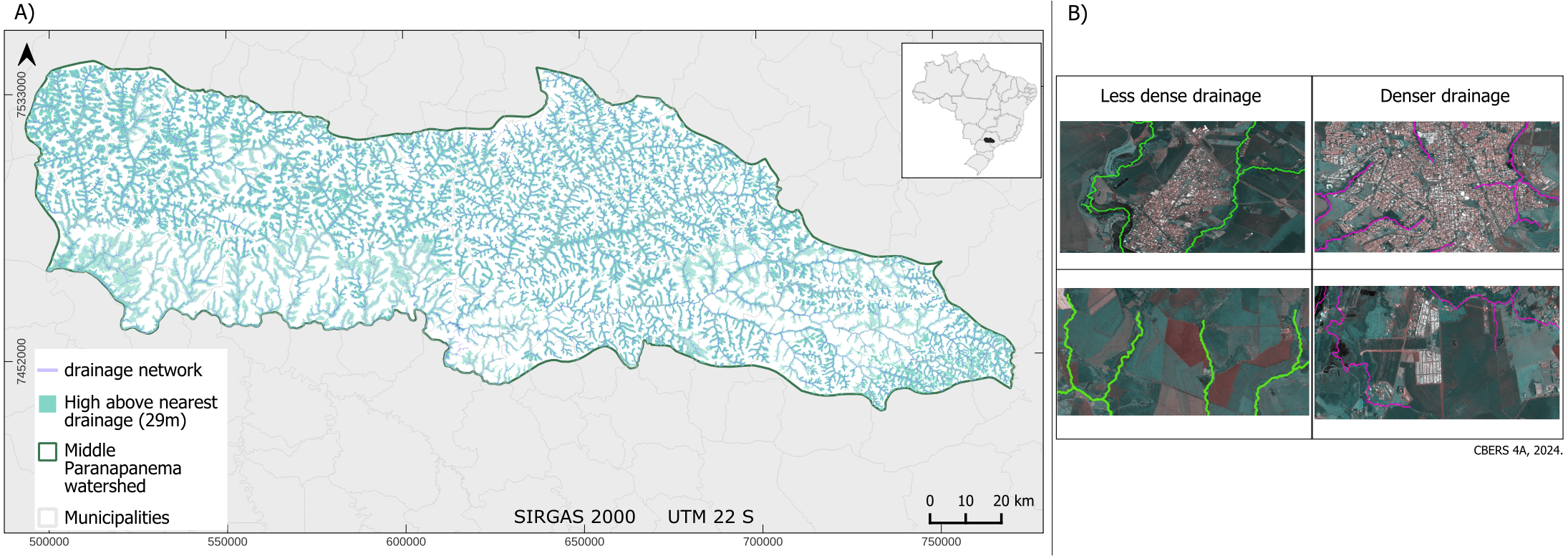

Supplement: S2 Fig — A) The map shows the outcome of the methodological strategies applied to identify the extent of wetland areas within the MP basin. It illustrates drainage network and high above nearest drainage (HAND) as indicators of freshwater presence. The wetland areas identified, primarily located surrounding the drainage network, cover 43.5% of the entire basin’s area. B) The satellite image shows the difference between the two drainage network in different areas. The ultimate drainage network was constituted by the amalgamation of these two components, with its configuration influenced by the topographical features. Municipality border shape available from https://geoftp.ibge.gov.br/organizacao_do_territorio/malhas_territoriais/malhas_municipais/municipio_2022/Brasil/BR/BR_Municipios_2022.zip. Terms of use available from https://biblioteca.ibge.gov.br/visualizacao/livros/liv101998.pdf. Satellite image available from http://www.dgi.inpe.br/catalogo/explore. Terms of use available from https://www.gov.br/pt-br/servicos/obter-imagens-de-sensoriamento-remoto-da-terra-geradas-pelo-satelite-cbers-04a. MP border shape available from https://datageo.ambiente.sp.gov.br/geoserver/datageo/LimiteUGRHI/wfs?version=1.0.0&request=GetFeature&outputFormat=SHAPE-ZIP&typeName=LimiteUGRHI. License information available from https://datageo.ambiente.sp.gov.br/sobre. (TIF) [file pntd.0012582.s002.tif]

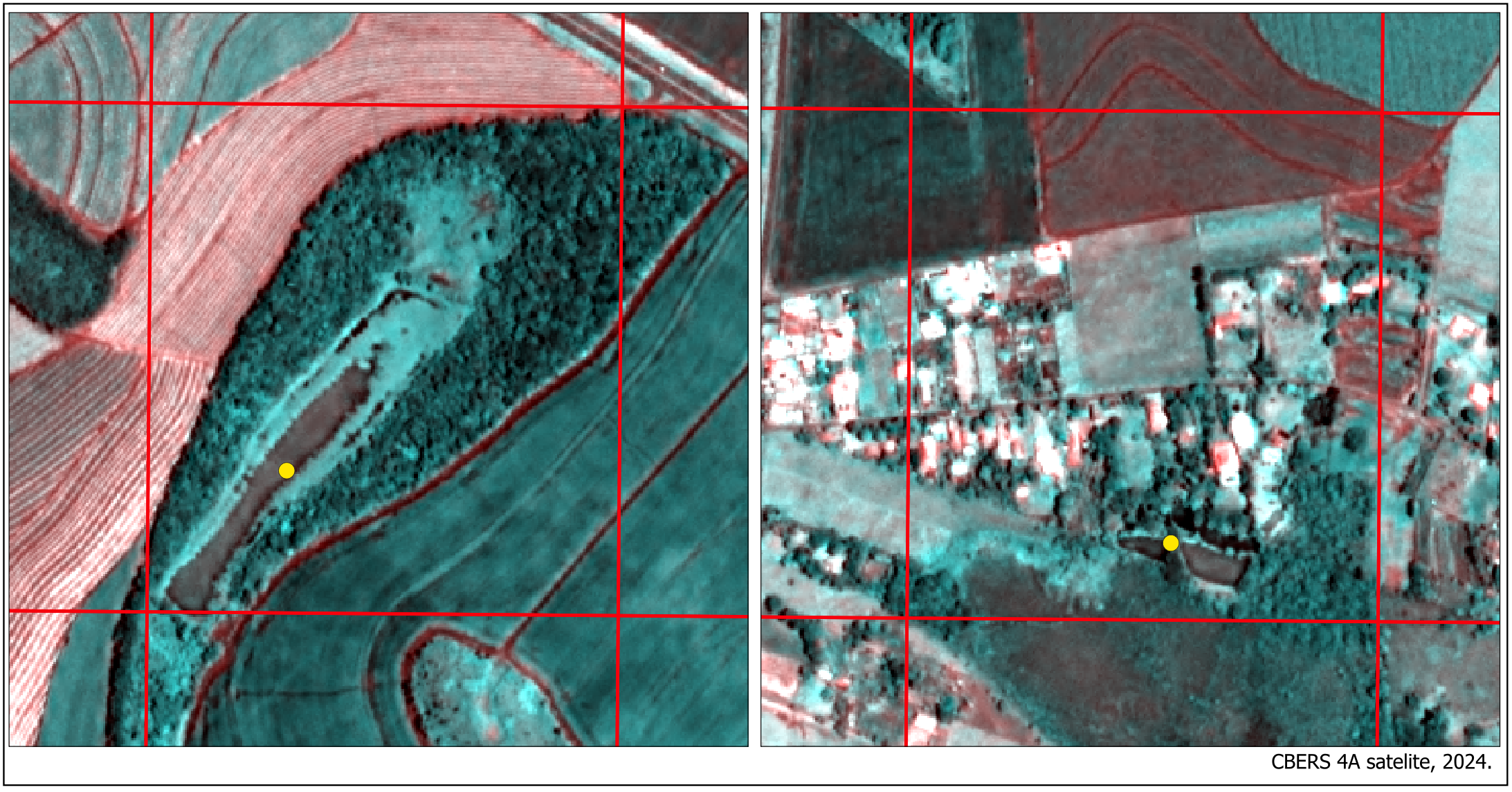

Supplement: S3 Fig — The figure shows the two B. glabrata in landscape unit Type 9 collection points near water bodies in cells with other land use and land cover. The classification rules, exemplified by parameters like the proportion of cell area occupied by forest vegetation or urbanized features, served to designate these areas as distinct from other landscape types. Satellite image available from http://www.dgi.inpe.br/catalogo/explore. Terms of use available from https://www.gov.br/pt-br/servicos/obter-imagens-de-sensoriamento-remoto-da-terra-geradas-pelo-satelite-cbers-04a. (TIF) [file pntd.0012582.s003.tif]
