# Supplementary material for: Mapping schistosomiasis risk landscapes and implications for disease control: A case study for low endemic areas in the Middle Paranapanema river basin, São Paulo, Brazil
Source: PLoS Negl Trop Dis. 2024 Nov 4;18(11):e0012582. doi: 10.1371/journal.pntd.0012582 (PMC11563476; doi:10.1371/journal.pntd.0012582)
Supplement: S1 Appendix — Description of the metrics employed in the analysis of individual polygons belonging to the three distinct classes at two levels: (i) at the class level (C) and (ii) at the landscape level (L). (PDF) [file pntd.0012582.s004.pdf]

S1 Appendix. Landscape Metrics Description - Presents the description of the metrics employed in the analysis of individual polygons belonging to the three distinct classes at two levels: (i) at the class level (C) and (ii) at the landscape level (L).

(C) Class level metrics

(C) Aggregation Index (AI): This metric quantifies the number of similar adjacencies involving the corresponding patch (polygon class). It is derived by dividing the actual count of similar adjacencies within the specific class by the highest potential count of similar adjacencies possible within that class. This maximum count occurs when the class is entirely clumped together into a single, compact patch. The resulting value is then multiplied by the proportion of the landscape that comprises the specific class. This process is repeated for all classes and the collective sum is multiplied by 100 to convert it into a percentage. The aggregation index is computed from an adjacency matrix at the class level. When patch types are at maximum dispersion (i.e., no similar adjacencies), AI equals 0. As the landscape becomes increasingly aggregated, AI increases in value, reaching 100 when the landscape is composed of a single patch, which may not necessarily be square in shape.

(C) PLAND, Proportion of Landscape, is determined by summing the areas (in square meters) of all patches (polygon units of a specific class) corresponding to that patch type. This sum is then divided by the total area of the landscape (measured in square meters) and multiplied by 100 to convert the value into a percentage. Essentially, PLAND signifies the percentage of the landscape occupied by the specific patch type. As the corresponding patch type becomes increasingly scarce within the landscape, PLAND approaches 0. When the entire landscape is dominated by a single patch type, PLAND equals 100, indicating that the entire grid comprises a singular patch type.

(C) Percentage of Like Adjacencies (PLADJ): PLADJ is calculated by dividing the number of similar adjacencies involving the focal class by the total number of cell adjacencies related to the focal class. The resulting value is then multiplied by 100 to express it as a percentage. In essence, PLADJ represents the proportion of cell adjacencies related to the specific patch type that are similar adjacencies. PLADJ equals 0 when the corresponding patch type is maximally dispersed (i.e., when each cell represents a different patch) and there are no similar adjacencies. This scenario occurs when the class is divided into individual one-cell patches. When the landscape is comprised of a single patch, and all adjacencies involve the same class, PLADJ equals 100.

(C) SPLIT: The Splitting index is calculated by dividing the square of the total landscape area (in square meters) by the sum of the squared patch areas (in square meters) across all patches of the corresponding patch type. When the landscape is composed of a single patch, SPLIT equals 1. As the area of the focal patch type decreases and is divided into smaller patches, SPLIT increases. The maximum value of SPLIT is bounded by the ratio of landscape area to cell size and is reached when the corresponding patch type is a single patch of one pixel.

(L) Landscape level metrics

(L) Mean Euclidean Nearest Neighbour Distance (ENN_MN): This metric serves as a measure of patch isolation. It quantifies the nearest neighbour distance by employing Euclidean geometry, which calculates the shortest straight-line distance between the focal patch and its nearest neighbour of the same class. This computation is based on the distance between the cell centers of the two closest cells belonging to the respective patches.
