# Supplementary material for: Mapping schistosomiasis risk landscapes and implications for disease control: A case study for low endemic areas in the Middle Paranapanema river basin, São Paulo, Brazil
Source: PLoS Negl Trop Dis. 2024 Nov 4;18(11):e0012582. doi: 10.1371/journal.pntd.0012582 (PMC11563476; doi:10.1371/journal.pntd.0012582)
Supplement: S1 Table — The table presents the 23 municipalities within the MP basin wherein the methodology applied for rectifying overlaps in the Cadastro Ambiental Rural (CAR) corrected 32,706 hectares of overlap. Each municipality is accompanied by information detailing the declared area in the CAR, the corrected area subsequent to rectification, and the corresponding percentage disparity between these two values. (PDF) [file pntd.0012582.s005.pdf]

S1 Table. CAR Corrected Table. The table presents the 23 municipalities within the MP basin wherein the methodology applied for rectifying overlaps in the Cadastro Ambiental Rural (CAR) corrected 32,706 hectares of overlap. Each municipality is accompanied by information detailing the declared area in the CAR, the corrected area subsequent to rectification, and the corresponding percentage disparity between these two values.

Municipality	Area declared in the CAR (ha)	Area corrected (ha)	Difference between values of ares (%)
Assis	36765.8	36704.4	-61.4 (-0.2%)
Avaré	113570	112723.2	-846.8 (0.7%)
Cabrália Paulista	26596	24345.8	-2250.2 (-8.5%)
Cerqueira César	48939.7	48860.4	-79.3 (0.2%)
Echaporã	52948.9	49596.3	-3352.6 (-6.3%)
Espírito Santo do Turvo	18675.7	18236.1	-439.6 (-2.4%)
Fernão	8962.26	8876	-86.26 (-1.0%)
Florínea	20102.5	20007.1	-95.4 (-0.5%)
Gália	34510.7	31006.1	-3504.6 (-10.2%)
Iaras	47631.4	31570	-16061.4 (-33.7%)
Itatinga	80134.8	80132.6	-2.2 (0%)
João Ramalho	47784.6	47729.9	-54.7 (-0.1%)
Lupércio	16334.5	16309.3	-25.2 (-0.2%)
Lutécia	48927.4	48921.2	-6.2 (-0%)
Maracaí	47994.3	47835.3	-159 (-0.3%)
Ocuaçu	26518.1	26501.5	-16.6 (-0.1%)
Óleo	20159.1	20088	-71.1 (-0.4%)
Paraguaçu Paulista	104525.8	104293.6	-232.2 (-0.2%)
Pardinho	20762.1	20594.9	-167.2 (-0.8%)
Paulistânia	20956	20468.9	-487.1 (-2.3%)
Quatá	64645.4	64424.4	-221 (-0.3%)
Rancharia	173468.5	169090.5	-4378 (-2.5%)
São Pedro do Turvo	74440.3	74332.4	-107.9 (-0.1%)
Total	1155353.86	1,122,647.9	-32,705.96 (-2.83%)
