# Supplementary material for: Mapping schistosomiasis risk landscapes and implications for disease control: A case study for low endemic areas in the Middle Paranapanema river basin, São Paulo, Brazil
Source: PLoS Negl Trop Dis. 2024 Nov 4;18(11):e0012582. doi: 10.1371/journal.pntd.0012582 (PMC11563476; doi:10.1371/journal.pntd.0012582)
Supplement: Attachment — Submitted filename: PNTD-D-24-00139 Reviewer Final.pdf [file pntd.0012582.s007.pdf]

PLOS Neglected Tropical Diseases

Mapping schistosomiasis risk landscapes and implications for disease control: a case study for low endemic areas in the Middle Paranapanema river basin, São Paulo, Brazil

--Manuscript Draft--

**Manuscript Number:**	PNTD-D-24-00139
**Full Title:**	Mapping schistosomiasis risk landscapes and implications for disease control: a case study for low endemic areas in the Middle Paranapanema river basin, São Paulo, Brazil
**Short Title:**	Schistosomiasis risk landscapes mapping in Middle Paranapanema river basis, SP, Brazil
**Article Type:**	Research Article
**Keywords:**	Schistosomiais; schistosomiasis surveillance; landscape; risk map; earth observation data
**Abstract:**	Background<br>Schistosomiasis, a chronic parasitic disease, remains a public health issue in tropical and subtropical regions, especially in low and moderate-income countries lacking assured access to safe water and proper sanitation. A 2011-2015 Brazilian survey found a decrease in human infection rates to 1%, with 19 out of 26 states still classified as endemic areas. There is a risk of schistosomiasis reemerging as a public health concern in low-endemic regions. This study proposes an integrated landscape-based approach to aid surveillance and control strategies for schistosomiasis in low-endemic areas.<br>Methodology/Principal findings<br>In the Middle Paranapanema river basin, specific landscapes linked to schistosomiasis were identified using a comprehensive methodology. This approach merged remote sensing, environmental, socioeconomic, epidemiological, and malacological data. A team of experts identified ten distinct landscape categories associated with varying levels of schistosomiasis transmission potential. These categories were used to train a supervised classification machine learning algorithm, resulting in a 92.5% overall accuracy and a 6.5% classification error. Evaluation revealed that 74.6% of collected snails from water collections in five key municipalities within the basin belonged to landscape types with higher potential for S. mansoni infection. Landscape connectivity metrics were also analysed.<br>Conclusions/Significance<br>This study highlights the role of integrated landscape-based analyses in shaping strategies for eliminating schistosomiasis. The methodology has produced new schistosomiasis risk maps covering the entire basin. The region's low endemicity might be partly due to the limited connection among grouped landscape-units more prone to triggering schistosomiasis transmission. Nevertheless, changes in social, economic, and environmental landscapes, especially those linked to the rising pace of incomplete urbanization processes in the region, have the potential to increase risk of schistosomiasis transmission. This study will help target interventions to bring the region closer to schistosomiasis elimination.
**Additional Information:**	
**Question**	**Response**
**Government Employee**<br><br>Are you or any of the contributing authors an employee of the United States government?	No - No authors are employees of the U.S. government.

Powered by Editorial Manager® and ProduXion Manager® from Aries Systems Corporation

Manuscripts authored by one or more US Government employees are not copyrighted, but are licensed under a CC0 Public Domain Dedication, which allows unlimited distribution and reuse of the article for any lawful purpose. This is a legal requirement for US Government employees.<br><br>This will be typeset if the manuscript is accepted for publication.	
**Financial Disclosure**<br><br>Enter a financial disclosure statement that describes the sources of funding for the work included in this submission. Review the submission guidelines for detailed requirements. View published research articles from *PLOS Neglected Tropical Diseases* for specific examples.<br><br>This statement is required for submission and **will appear in the published article** if the submission is accepted. Please make sure it is accurate.<br><br>**Funded studies**<br>Enter a statement with the following details:<br>• Initials of the authors who received each award<br>• Grant numbers awarded to each author<br>• The full name of each funder<br>• URL of each funder website<br>• Did the sponsors or funders play any role in the study design, data collection and analysis, decision to publish, or preparation of the manuscript?<br><br>Did you receive funding for this work?	Yes
Please add funding details.<br>as follow-up to "**Financial Disclosure**<br><br>Enter a financial disclosure statement that describes the sources of funding for the work included in this submission. Review the submission guidelines for detailed requirements. View published research	This study was funded by the Regular Research Project, Integrated risk mapping and targeted snail control to support schistosomiasis elimination in Brazil and Cote d'Ivoire under future climate change, Awarded n. 2019/23593-3, FAPESP-SP/Brazil Line of Funding associated with the BELMONT FORUM Cooperation Agreements. VAFS was funded by CAPES-Coordenação de Aperfei¸coamento de Pessoal de Nível Superior, Ministry of Education of Brazil (CAPES, Finance Code 001) and the AEB - Brazilian Space Agency, with a Master's Scholarship for the graduate programme in Remote Sensing and Geoinformation at INPE. Field work was partially funded by the SUCEN

articles from *PLOS Neglected Tropical Diseases* for specific examples.<br><br>This statement is required for submission and **will appear in the published article** if the submission is accepted. Please make sure it is accurate.<br><br>**Funded studies**<br>Enter a statement with the following details:<br>• Initials of the authors who received each award<br>• Grant numbers awarded to each author<br>• The full name of each funder<br>• URL of each funder website<br>• Did the sponsors or funders play any role in the study design, data collection and analysis, decision to publish, or preparation of the manuscript?<br><br>Did you receive funding for this work?"	division incorporated by the Pasteur Institute of the Secretary of Health of the State of São Paulo. MK has been partially funded by grants from UKRI-GCRF 723 (EP/T003820/1), FAPESP (2021/04128-8), and FUSP (2017/00686-0). AS, CG, AC and GL were partially funded by the Belmont Collaborative Forum for Climate, Environment and Health and the US National Science Foundation.
Please select the country of your main research funder (please select carefully as in some cases this is used in fee calculation).<br>as follow-up to "**Financial Disclosure**<br><br>Enter a financial disclosure statement that describes the sources of funding for the work included in this submission. Review the submission guidelines for detailed requirements. View published research articles from *PLOS Neglected Tropical Diseases* for specific examples.<br><br>This statement is required for submission and **will appear in the published article** if the submission is accepted. Please make sure it is accurate.	BRAZIL - BR

Powered by Editorial Manager® and ProduXion Manager® from Aries Systems Corporation

**Funded studies**<br>Enter a statement with the following details:<br>• Initials of the authors who received each award<br>• Grant numbers awarded to each author<br>• The full name of each funder<br>• URL of each funder website<br>• Did the sponsors or funders play any role in the study design, data collection and analysis, decision to publish, or preparation of the manuscript?<br><br>Did you receive funding for this work?"	
**Competing Interests**<br><br>On behalf of all authors, disclose any competing interests that could be perceived to bias this work.<br><br>This statement will be typeset if the manuscript is accepted for publication.<br><br>Review the instructions link below and PLOS NTDs' competing interests policy to determine what information must be disclosed at submission.	The authors declare that the research was conducted in the absence of any commercial<br>or financial relationships that could be construed as a potential conflict of interest.
**Data Availability**<br><br>Provide a **Data Availability Statement** in the box below. This statement should detail where the data used in this submission can be accessed. This statement will be typeset if the manuscript is accepted for publication.<br><br>Before publication, authors are required to make all data underlying their findings fully available, without restriction. Review our PLOS Data Policy page for detailed information on this policy. Instructions for writing your Data Availability statement can be accessed via the Instructions link below.	The raw data supporting the conclusion of this article will be made available by the authors, without undue reservation.

Powered by Editorial Manager® and ProduXion Manager® from Aries Systems Corporation

Manuscript Click here to access/download;Manuscript;Manuscript.pdf

Mapping schistosomiasis risk landscapes and implications for disease control: a case study for low endemic areas in the Middle Paranapanema river basin, São Paulo, Brazil

Vivian Alessandra Ferreira da Silva*[1], Milton Kampel[1,2], Rafael Silva dos Anjos[3], Raquel Gardini Sanches Palasio[4], Maria Isabel Sobral Escada[1,3], Roseli Tuan[5], Alyson L. Singleton[6], Caroline Glidden[7], Andy Chamberlin[8], Giulio A. De Leo[8,9], Adriano Pinter[5], Antônio Miguel Vieira Monteiro*[1,3]

1 Remote Sensing and Geoinformatics Graduate Program. Earth Observation and Geoinformatics Division (DIOTG), National Institute for Space Research (INPE), São José dos Campos, SP, Brazil
2 MOceanS - Monitoring Oceans from Space Laboratory. DIOTG-INPE
3 LiSS –Laboratory for investigation of Socio Environmental Systems. DIOTG-INPE
4 Laboratory of Spatial Analysis in Health (LAES), Department of Epidemiology, School of Public Health, University of Sao Paulo (FSP/USP), São Paulo, SP, Brazil
5 Pasteur Institute, São Paulo, SP, Brazil
6 Emmett Interdisciplinary Program in Environment and Resources, Stanford University, Stanford, CA, USA
7 Department of Biology, Stanford University, Stanford, CA, USA
8 Department of Oceans, Hopkins Marine Station, Stanford University, Pacific Grove, CA, USA
9 Woods Institute for the Environment, Stanford University, Stanford, CA, USA

* vivianafdsilva@gmail.com, miguel.monteiro@inpe.br

Abstract

Background

Schistosomiasis, a chronic parasitic disease, remains a public health issue in tropical and subtropical regions, especially in low and moderate-income countries lacking assured access to safe water and proper sanitation. A 2011-2015 Brazilian survey found a decrease in human infection rates to 1%, with 19 out of 26 states still classified as endemic areas. There is a risk of schistosomiasis reemerging as a public health concern in low-endemic regions. This study proposes an integrated landscape-based approach to aid surveillance and control strategies for schistosomiasis in low-endemic areas.

Methodology/Principal findings

In the Middle Paranapanema river basin, specific landscapes linked to schistosomiasis were identified using a comprehensive methodology. This approach merged remote sensing, environmental, socioeconomic, epidemiological, and malacological data. A team of experts identified ten distinct landscape categories associated with varying levels of schistosomiasis transmission potential. These categories were used to train a supervised classification machine learning algorithm, resulting in a 92.5% overall accuracy and a 6.5% classification error. Evaluation revealed that 74.6% of collected snails from water collections in five key municipalities within the basin belonged to landscape types with higher potential for *S. mansoni* infection. Landscape connectivity metrics were also analysed.

Conclusions/Significance

This study highlights the role of integrated landscape-based analyses in shaping strategies for eliminating schistosomiasis. The methodology has produced new schistosomiasis risk maps covering the entire basin. The region's low endemicity might be partly due to the limited connection among grouped landscape-units more prone to triggering schistosomiasis transmission. Nevertheless, changes in social, economic, and environmental landscapes, especially those linked to the rising pace of incomplete urbanization processes in the region, have the potential to increase risk of schistosomiasis transmission. This study will help target interventions to bring the region closer to schistosomiasis elimination.

Author summary

Schistosomiasis is a Neglected Tropical Disease whose transmission in Brazil is related to human contact with water contaminated by the trematode parasite *Schistosoma mansoni* Sambon, 1907. The national prevalence survey from 2011-2015 revealed a decline in schistosomiasis positivity rates to 1%, marking areas in the Middle Paranapanema river basin in SP-Brazil (MP) as low endemic. However, control programs face additional challenges due to the new dynamics connected with the social, environmental, and economic developments fueled by an urban-industrial urbanization model in the MP region. To address this, our study proposes a landscape-based methodological approach that categorizes and assesses schistosomiasis transmission potential in different regional landscape patterns. By combining remote sensing, environmental, socioeconomic, epidemiological, and malacological data, a multidisciplinary team identified ten distinct landscape-unit types associated with varying levels of schistosomiasis transmission potential. Using a decision-tree based machine learning classification method, we mapped schistosomiasis risk across the basin at a landscape scale. The spatial identification and characterization of the current landscape situation aid in devising suitable surveillance and control strategies at both local and regional levels. Our findings indicate that landscape-units with higher transmission potentials are less interconnected across the basin. Low connectivity at the landscape scale, in part, explains the MP low endemic areas, contributing to the low endemicity in the Middle Paranapanema region. This approach offers promise for local and regional schistosomiasis management efforts aimed at eradicating the disease.

Introduction

Schistosomiasis is a Neglected Tropical Disease (NTD) and one of the most widespread parasitoses in the world, registered in Africa, Asia, the Caribbean and South America. The World Health Organization (WHO) Fact sheets for schistosomiais has estimated that, at least, 251.4 million people required preventive treatment in 2021 [1]. Human schistosomiasis is an acute and chronic water-based disease that presents itself in two major forms, intestinal and urogenital, and it is prevalent in tropical and subtropical areas, especially in low and moderate-income countries lacking assured access to safe water and proper sanitation. It is caused by parasitic trematodes from the genus *Schistosoma* which has 5 of its main species displaying distinct geographical distributions around the globe. The parasite matures within aquatic snails, acting as intermediate hosts until it transforms into a stage called cercariae, capable of infecting humans who come into contact with contaminated water sources. The etiologic agent in Brazil is the *Schistosoma mansoni* Sambon, 1907, where three species of snails of the genus *Biomphalaria* have been found to be naturally infected: ***B. glabrata*** (Say, 1818), ***B. straminea*** (Dunker, 1848) and ***B. tenagophila*** (Orbigny, 1835). These snails are

widespread throughout the country [2].

The initial cases of schistosomiasis were documented in Brazil in 1908. In the years that followed until the early 1930s, investigations into the geographical distribution of the disease intensified and clinical, parasitological and malacological data were accumulated, showing that schistosomiasis was an important public health problem. In the 1950s, schistosomiasis was consolidated as an endemic disease in Brazil, mainly prevalent in regions characterized as rural areas [3, 4]. The Schistosomiasis Control Programme (PCE), initiated in Brazil in 1975, introduced uniform and integrated measures across the country, focusing on the reduction of disease prevalence and its morbidity in endemic areas by employing praziquantel treatment and molluscicide (Bayluscid) application in freshwater collections with *S. mansoni*-infected snails.

The prevalence of the disease has significantly declined from double-digit figures in the 1950s to a range of 1% to 5% in recent years. This indicates the effectiveness of the Schistosomiasis Control Program (PCE) strategy and the advancements in access to water, sanitation, and hygiene (WASH) in Brazil in altering the disease's prevalence [5, 6]. However, the risk of schistosomiasis transmission persists in specific geographical areas, now, different from the past, often situated near urban areas or peri-urban zones. These new areas, considered urbanised, and the zones between these areas of greater concentration and density and the rural areas, with lower densities and concentrations, which we refer to here as peri-urban areas, are spaces characterised by an incomplete urbanisation where production spaces and housing are established, but with inadequate access to facilities and services that would prevent the contamination of water collections by infected snail [7–11].

A substantial shift in the distribution patterns of *B. straminea*, *B. glabrata*, and *B. tenagophila* was documented in a malacological investigation conducted within freshwater collections situated in the middle section of the Paranapanema river basin in Sao Paulo, Brazil [12]. This shift highlights fluctuations in the capacity of specific habitats to sustain these species. But what potential impact could these changes have on disease transmission? Considering that each neotropical host exhibits distinct susceptibility to the parasite [13], this observation could imply a potential adjustment in the risk of local human infection.

Additionally, the three species display varying responses to changes in surface water temperature [14]. As the global temperature continues to rise, there is a likelihood of an increase in the frequency and intensity of extreme weather events. Changes in water temperature, shifts in rainfall patterns, and the occurrence of severe flooding leading to the expansion of flooded areas could be linked to the emergence of this new climate stage. This new situation brings new challenges for the fight against schistosomiasis [15, 16]. Furthermore, land use and land cover changes have transformed the middle section of the Paranapanema river basin at the landscape scale, impacting the basin's drainage system throughout the period of human occupation. This process has continued in recent years. These circumstances introduce fresh dynamics within the basin's waterways, presenting novel prospects for linking freshwater habitats that host these species with those where they were previously absent. This scenario remains relatively little explored in current literature [17–19].

Two recent WHO documents dealing with neglected tropical diseases in general and schistosomiasis in particular, put the elimination of schistosomiasis as a public health problem at the centre of the agenda [20, 21, 21]. We are moving forwards, but the fact is that mass administration of praziquantel (MDA) remains today the main control and elimination strategy. There is also an important initiative in the direction of the development of a safe formulation of this chemotherapy drug that can be administered to children aged 3 months to 6 years which is progressing rapidly [23]. Also, although there have been good advances in WASH strategies, education and communication

actions about the disease, what we still see is an uneven distribution of these improvements across the different regions of the planet [5].

For areas of low endemicity, such as the Middle Paranapanema basin, these new scenarios of possible changes in rainfall and temperature patterns associated to changes in the patterns of movements of people and goods, pose major challenges for schistosomiasis control and elimination programmes in the wake of advances in the processes of a metropolitan model of urbanisation in the state of São Paulo towards small and medium-sized cities. Particularly in these areas, if the elimination of schistosomiasis on a local scale is a goal to be achieved, it is essential to think of new strategies for mapping areas at risk of potential transmission that can guide targeted intervention actions. However, it is the social, economic, environmental and climatic dynamics that transform the inhabited and uninhabited landscapes in the basin areas. In these transformed and changing landscapes, the relationships and interdependencies that snails, humans and parasites have with each other are also altered to varying degrees. Therefore, thinking about control and elimination in situations of low endemicity requires a focus on a geographic region of action and the characterization of its specific landscapes for the contexts involving schistosomiasis and its transmission. However, considering the connectivity of these local landscapes, the relationship of these landscapes with other schistosomiasis landscapes on a regional scale is essential. To address schistosomiasis low endemic areas transmission potential risk mapping oriented towards surveillance and control systems involved in strategies for disease control and elimination programs, our study proposes a landscape-based method approach [24, 25].

Our study fits within a methodological framework that, first, categorizes and assesses schistosomiasis transmission potential for different regional landscape patterns. In our approach, a set of schistosomiasis landscape patterns was defined by a multidisciplinary team, which combined local knowledge and literature with orbital remote sensing images, environmental, socioeconomic, epidemiological and malacological data, and identified and characterized ten distinct types of landscape units associated with different levels of schistosomiasis transmission potential in the region of the Middle Paranapanema river basin. These ten landscape units form a set of types for schistosomiasis landscapes in the region. Together, these types define the typology of schistosomiasis landscapes at the basin scale. These types of landscape were used to train a supervised classification machine learning algorithm. Using a decision-tree based classification method, we mapped schistosomiasis risk across the basin at a landscape scale. The whole area of the Middle Paranapanema river basin was represented by a grid of [500x500] meters cells. Each cell represented a type of landscape unit that should be labeled by the classification algorithm. Our findings indicate that landscape-units with higher transmission potentials are less interconnected across the basin. Low connectivity, in part, explains the basin´s low endemic areas, contributing to the low endemicity in the Middle Paranapanema region. For an agenda that targets the elimination of the schistosomiasis as a public health problem, it would be highly important that surveillance and control systems integrate into its analytic toolbox methods that combine the new methodologies with more established ones [26]. Our approach offers promise for local and regional schistosomiasis management efforts that seek its elimination by 2030 by promoting surveillance-response systems instruments that are more tailored to the social- ecological settings found in the basin region.

Methodology

Study area

The Middle Paranapanema river basin (MP), located in Southeast, Brazil, includes 55 municipalities. The region stands out among the endemic sites for schistosomiasis in the state of São Paulo, Brazil, with a transmission history since 1952. The first autochthonous cases occurred in the municipalities of Ourinhos, Ipaussu and Palmital [27, 28]. Eight out of 55 municipalities in the MP region have reported autochthonous cases of schistosomiasis. The last autochthonous cases in the MP region occurred in the municipalities of Ourinhos (2018), Ipaussu and Ribeirão do Sul (2017), Chavantes and Santa Cruz do Rio Pardo (2016), Palmital (2011) and Canitar (2008) [29]. Although Assis had the second-highest number of cases within the historical record, trailing only behind Ourinhos, the occurrence of its last autochthonous cases dates back to 1997 [30]. These eight municipalities and another 17 municipalities in the MP basin are included as geosentinel units and are prioritised for schistosomiasis monitoring and control by the Epidemiological Surveillance Groups (GVE). The GVEs are part of the surveillance and control structure of the São Paulo state health department, and work with national programmes [31]. For the region of this study, the municipalities with autochthonous cases are part of the geosentinel units associated with GVE-13 (GVE Assis). The total population of the MP basin was 1,320,973 inhabitants in 2022 (IBGE), and the population of the eight municipalities with registered autochthonous cases was 302,316 inhabitants [32]. Epidemiological data on schistosomiasis cases in the MP basin region are available by the year of onset of symptoms (or by the year of notification of the disease) and aggregated at municipal level. The case database covers the period from from 2006 to 2022 [32]. Fig 1 shows a map of the MP basin and highlights both the eight municipalities that have reported autochthonous cases and the aggregated number of schistosomiasis cases for the municipalities in the basin between 1976 and 2022 (or 2006 and 2022).

Fig 1. The Middle Paranapanema river basin, São Paulo, Brazil, highlighting eight municipalities that have had autochthonous cases at some point and the aggregate of schistosomiasis cases by year of the onset of symptoms for the 55 municipalities in the basin in the period between 2006 and 2022.

Data and methods

Fig 2 illustrates a concise schematic diagram detailing the fundamental procedural stages necessary for producing the schistosomiasis landscape risk maps. Within the context of the MP basin, the employed datasets are presented, in particular, regarding malacological and landscape characterization data. The methodology section delineates the process involved in constructing the typology of landscape units associated with schistosomiasis within the basin. Additionally, it elaborates on the methods employed to create the essential variables required for the classification process using machine learning techniques. Finally, it outlines how these variables were employed in a supervised classification strategy, utilizing decision tree methods.

Fig 2. Simplified general schematic diagram of methodology.

Malacological data

A comprehensive investigation into *Biomphalaria* identification was carried out between 2014 and 2018, sweeping a large set of water collection, across five municipalities that reported autochthonous cases: Ourinhos, Ipaussu, Ribeiro do Sul, Chavantes, and Assis. The database relating to this collection, with the identification and localisation of three species of intermediary host snail, *B. glabrata*, *B. tenagophila* and *B. straminea*, has been used in this study [33]. Fig 3 shows their geographical distribution.

Fig 3. *B. glabrata*, *B. tenagophila* and *B. straminea* geographical distribution in five municipalities of the MP basin: Ourinhos, Ipaussu, Ribeirão do Sul, Chavantes, and Assis.

Landscape characterisation data

Physical landscape data

The land use and land cover (LULC data) utilized in this study was derived from the MapBiomas project [34]. The classes of agriculture, pasture, urbanised areas and forest fragments (Atlantic forest) have helped to delineate landscapes with economic activity and human presence at different levels of potential contact with host snails, landscapes with a strong and more dense human presence and spaces with few chances of encounter between snails and humans respectively. Despite the inherent constraints of the 30-meter spatial resolution (Landsat images are used by MapBiomas), which may hinder the precise identification of targets demanding finer details, it proved suitable for the landscape scale applied in this particular investigation.

The roads database provided by Open Street Map (OSM) [35] was reclassified according to a Mobility Typology [36]. This reclassification facilitated the rapid identification of road types—whether local or regional—and their respective travel speeds. Distinguishing between local roads, residential pathways, non-automotive routes, and potential forthcoming infrastructural developments aided in pinpointing landscape types that exhibit higher potential for human-parasite interaction.

Phase Array L-band Synthetic Aperture Radar (PALSAR) imagery from the Advanced Land Observing Satellite (ALOS) [37] was used to derive several key elements. These included the relief-adjusted drainage network, High Above Nearest Drainage (HAND) calculations [38], and the assessment of the slope along the edges of water bodies. The generation of the relief-adjusted drainage network took into account the landforms delineated in the cartographic base of geomorphological units in Brazil [39]. This drainage network was created using an open source and free software, the TerraHidro platform [40], employing dual thresholds tailored to regions characterized by either high or low densities of water channels. Subsequently, the slopes along the margins of water bodies were computed based on the PALSAR/ALOS image mosaic associated with the relief-adjusted drainage network.

The High Above Nearest Drainage (HAND) method leverages the directional flow of nearby watercourses to compute the vertical distance from a specified location to the closest point on the drainage network. This set of nearest vertical distances was taken to identify regions contiguous to watercourses as potential habitats for intermediate host snails, drawing upon malacological data. Unlike "buffer" zoning operations which focus on horizontal distances between a point and the drainage, HAND accounts for terrain altitude fluctuations, offering a more nuanced assessment of topographical variations.

Socio-demographic landscape data

The Brazilian Demographic Census survey data provides information on the situation of the households and their surroundings aggregated by spatial units called census tracts. The aggregated information provides data on the situation of the census tract, whether urban or rural, the number of households and the situation of access to water and sanitation in the sector. The last census with disaggregated information at census tract level was the 2010 demographic census [41], so this information was used. However, there is an updated sector mesh for the 2022 census [42] which only gives information on the sector's situation (urban or rural). This information, updated for 2022, better describes the urban/rural environment in the basin region today and was therefore used in this study.

The Rural Environmental Registry (CAR) stands as a mandatory national registry encompassing all rural properties in Brazil. Functioning as a self-declaratory system, its primary objective is to compile an integrated repository of information regarding rural properties and their alignment with the country's environmental data [43]. Within this study, CAR data served as an essential auxiliary information for refining the land use and land cover data class related to agricultural activities, based on the size of the declared rural property in the MP basin region. Notably, identifying smallholdings predominantly engaged in small-scale agriculture, relying on manual labour directly involved in land cultivation, helps to characterise landscapes where the potential for contact between humans and the intermediate host snails of schistosomiasis. Conversely, identifying medium and large rural properties, where production involves a high degree of mechanisation and the use of chemical pesticides, where the way of producing involves a high degree of mechanisation, the use of chemical pesticides and fewer rural workers, establishes landscapes with less potential for encounters between humans and snails. In order to use the CAR database, it was necessary to use a methodology to correct overlaps and to define property sizes [44].

Information on the presence of domestic effluents discharge was derived from the application of a methodology based on the probability of human waste concentration in the drainage network. For modelling the possibility of domestic effluents in the drainage system, we used the Digital Elevation Model of the ALOS sensor on board the PALSAR satellite, as well as data from the 2010 Demographic Census of the Brazilian Institute of Geography and Statistics (IBGE) and the location of sewage treatment plants provided by the National Water and Basic Sanitation Agency (ANA). The estimate of water pollution was based on the relationship between the number of inhabitants per census tract associated with the nearest drainage. Drainages without the presence of domestic effluents were considered to be rivers and streams that had census tracts without inhabitants or were downstream from sewage treatment plants and, during their course, did not receive any discharge from a river or stream with the potential presence of domestic effluents [45]. Identifying water bodies with untreated effluent discharges helps to indicate the areas most likely to be infected with *S. mansoni* by intermediate host snails.

The mosaic of land use classes present in the land use and land cover database is a map that contains a mixture of agricultural uses related to small patches with vegetable production (e.g. lettuce, which is very common in the region), vegetable gardens and dirty pastures. Due to the 30 m spatial resolution used by the MapBiomas project, areas that cannot be properly classified into a specific type of agricultural production are included in this class, which states the presence of a mosaic of diversified small-scale agricultural uses.

In an approach based on the empirical exploration of data in the basin region, it was verified that 87% of autochthonous cases of schistosomiasis, reported between 1979 and 2016, were concentrated within a range of up to 225 meters from the contact areas between urbanised areas and the mosaic of LULC class, in Ourinhos, the only

municipality where finer spatial resolution cases data were available. This threshold value was defined based on the relationship between reported cases and the LULC map corresponding to the mid-decade period related to the year of case notification. The threshold corresponds to the maximum distance between the boundaries of the urbanized areas and the aggregate cases of schistosomiasis per household, at census tract level, in the period 1979-2016 [29]. Using this threshold, a contact zone between urbanized areas and the land use class mosaic of uses were defined using buffers of 225 m from the polygons of the urbanized areas. The contact zones were obtained by clipping the areas defined by these buffers, observing whether there was contact in the buffer area with the mosaic of uses LULC class.

The data incorporated in this study (Table 1) were specifically chosen for their capacity to elucidate and delineate the various landscape typologies potentially linked with schistosomiasis within the research area.

Table 1. Type of information, derived variables/index, data sources, and the respective year of reference of the databases used in this study. Data sources include IBGE (Brazilian Institute of Geography and Statistics), SICAR (National Rural Environmental Registry System), PALSAR/ALOS satellite data, and MapBiomas (annual land use and land cover maps across Brazil).

Information	Derived variable/Index	Data source	Year of reference
Land use and land cover (LULC) classification	LULC classes; contatc between the urbanized area LULC class and the mosaic of uses LULC class	MapBiomas	2022
Situation of the census tract	Urban/Rural	IBGE	2022
Households	Household's presence	IBGE	2010
Agricultural properties	Presence of agricultural production in small rural properties / Presence of agricultural production in medium or large rural properties	SICAR	2021
Roads	Types of roads associated to a mobility profile	Open Street Map	2021
Edges slope	Slope of water body edges	PALSAR/ALOS images	2011
Drainage network	Drainage network; High above nearest drainage (HAND) and water body edges	PALSAR/ALOS images	2011
Water without proper treatment	Sections of the basin's drainage network with the presence of domestic effluent discharge.	IBGE, PALSAR/ALOS images	2011

Typology building

Initially, in order to analyze schistosomiasis at the MP basin landscape scale, a multidisciplinary team delineated a set of schistosomiasis landscape patterns. These patterns were established based on the correlation observed between the constituents of the landscape being studied and their spatial arrangement in relation to the components involved in the transmission cycle of schistosomiasis. This team leveraged local expertise, field-gathered information, relevant literature, orbital remote sensing imagery, as well as environmental, socioeconomic, and malacological data to fully understand the landscape structures linked to schistosomiasis. These datasets were examined at fine

spatial resolutions, some of which included geographical coordinates acquired via GPS. Epidemiological data on cases and incidence, available solely at the municipal level, were incorporated as supplementary information to characterize this set of landscape types. This set of types establishes a typology for the schistosomiasis landscape units patterns in the context of the MP basin. The typology seeks to represent the ecological characteristics of the intermediate snails hosts' habitat together with features related to socio-territorial aspects of the human population living in the MP basin region.

Based on the joint analysis carried out by the multidisciplinary team using field experience and the collected dataset made available in an integrated environment using geographic information system (GIS), ten distinct landscape-unit types associated with varying levels of schistosomiasis transmission potential in the MP basin were identified. The complexity of schistosomiasis transmission, which involves an etiological agent, intermediate host, and definitive host, was considered in the typology. The built typology indicates the variables that can describe the particularities of schistosomiasis landscape types and bases the landscape classification process on the scale of the MP basin.

Types 1 and 2 represent environments that are more prone for the establishment of the transmission cycle of schistosomiasis as they combine important elements that favour contact between the etiologic agent, the intermediate host, and the definitive host. Types 3 and 4 bring together important characteristics that support the fixation of intermediate host snails, especially the availability of freshwater. Although the areas of these types may support the development of human activities, they do not have a discharge of untreated domestic effluents into water bodies, which makes the landscapes less prone to contamination by *S. mansoni*. Types 5 to 10 are not prone to the settlement of intermediate host snails and, except for Type 10, are not characterized by intense and abundant human presence. In Types 5, 6 and 7, the proximity of drainage is insufficient to provide life support for the mollusks. In Types 8, 9 and 10, the absence of fresh surface water and the distance from water bodies also prevent the presence of *S. mansoni* and the snails *B. glabrata*, *B. straminea* and *B. tenagophila*.

The final typology built on the basis of these procedures can be seen in the supporting information material (S1 Fig) where each schistosomiasis landscape unit is characterised and the rationale for its definition is explained. In Figure 4 we can see an example for a full description of a landscape-unit of Type 1.

Fig 4. A Type 1 landscape-unit of potential contact between humans and snails that may be infected. The landscapes described by this type involve urban and peri-urban areas close to lentic streams with highly favourable habitats for the reproduction of *Biomphalaria* sp., being small family farms with traditional vegetable cultivation, mainly lettuce, cultivated on flat or slightly sloping, deep, medium-textured soil (sandy-clay or sandy loam), aerated, supplied by a source/spring of water, well drained but with a reasonable water retention capacity, rich in organic matter and with sanitation deficiencies.

Landscape gridded representation

The entire extent of the MP basin was represented as a grid of 500x500-meter cells, forming a standardized grid where each cell denotes a distinct landscape unit to be identified and characterized. The selection of cells at this spatial resolution was guided by empirical testing aimed at assessing how the features of objects present in diverse and heterogeneous databases were portrayed at different cell resolutions. Cells of 100x100, 500x500, and 1000x1000 meters were tested to explore this representation.

Considering the limitations in spatial resolution inherent in the databases used in this study, the 500x500-meter resolution emerged as the most suitable in preserving the diversity of structures essential for identifying the landscape units associated with the types described in the typology. With 500x500 metre cells, 73.698 cells were used to cover the entire area of the MP basin.

A set of variables was incorporated into each of the regular grid cells. These variables were derived using different operators which made it possible to integrate information from different data sources with different formats (vector and matrix) and spatial resolutions (Table 2). In this way, it was possible to obtain a disaggregated distribution of the typology's variables, avoiding the problems of spatial non-coincidence between the data representation units. This approach made it possible to create a temporal stability of the units of analysis, aggregated by ad-hoc statistical or computational summarisation measures. This procedure is known as cell filling and, in this work, was carried out using the module in the free and open source TerraView 5.6.1 GIS software [46, 47].

Table 2. Landscape grid cell filling operators and the rationale for the inclusion of the variables.

Variable	Filling operation	Rationale for inclusion
Land Use and Land Cover classes	Percentage of each class	Land use and land cover classes allows to measure the observed landscape and the types of human activities (e.g. agriculture). Land use and land cover classes show elements of the physical landscape that can make up the construction of snail habitats. They help to characterise landscapes from a gradient perspective for potential contact situations between humans and snails.
Urbanised areas found to be close to areas of the LULC class mosaic of uses	Presence	The mosaic of land use and land cover classes show areas where there is agricultural production on small properties, small areas of secondary vegetation, small areas of clean and dirty pasture, in other words, a class that mixes some types of uses associated with activities carried out by small producers. Generally close to urbanised areas (urban and peri-urban areas). These areas, when present, represent a potential for contact between humans and snails, which may be infected.
Situation of the census tract	The situation of the census tract, urban or rural, that was most frequent in each cell. And the situation of the census tract, urban or rural, whose area had the greatest intersection with the area of the cell	Characterisation of the landscape according to their density of occupation, referred to by their urban or rural characterisation.
Households	Presence	Presence of households within that landscape unit indicates humans living and circulating in that landscape.
Roads according to the Mobility Typology classification	Maximum value	The higher the value assigned to the mobility class within the mobility typology, the more the street is characterized as a pathway for slower and localized movements.
Micro-size farms and small rural properties	Presence	Refinement of the agricultural land use class to improve the identification of small-scale agricultural activities.
Medium or large production properties	Presence	Refinement of the agricultural land use class to improve the identification of medium and large-scale agricultural activities.
Slope at the edge of water bodies	Maximum value	It indicates the slope situation at the edges of water bodies in water collections.
Relief-adjusted drainage network	Presence	Indirect characterisation of areas with potential for flooded areas and with conditions for the existence of snails.
Water without proper treatment	Presence	Stretches of water in the drainage network that receive untreated domestic effluent. This increases the potential for parasites in these stretches of river present in these landscape units.
HAND	Minimum value	The minimum HAND value identifies flooded (or floodable) areas where the conditions exist for finding the snails of interest.
Edges of water bodies	Presence	The edges of water bodies in water collections identify places where the snails of interest can be found.

Schistosomiasis landscape risk maps

Map of potential landscapes for Schistosomiasis: classification and assessment

The variables in Table 2 were allocated to each grid cell representing the landscape units of the MP basin. Together they form a vector of attributes associated with each 500x500 meter cell. This set of attribute vectors is used to apply a classification method using supervised machine learning based classification techniques. The landscape classification according to the typology defined was performed by a boosted decision tree classification method. The Boosted Decision Tree classifier is an ensemble method that combines multiple decision trees to enhance its predictive accuracy. This method iteratively constructs weak learners (decision trees), focusing on misclassified instances from the preceding iterations. Subsequent trees are adjusted to prioritize these misclassifications, iterating until the model achieves a better overall performance. Boosting, based on an adaptive learning method, aims to enhance predictive capability by diminishing both bias and variance in the model [48, 49]. The C5.0 decision tree algorithm [50], implemented in the Geographic Data Mining Analyst (GeoDMA) [51], was used to perform the classification.

Since it is a supervised method, the decision tree classification requires the use of samples provided by the analyst to train the classifier and assess the classification's quality. The boosting procedure generated 300 decision trees, decreasing the possibility of error in the classification process by seeking a more accurate result combining multiple iterations between the variables among all these trees. Among the 73,698 cells, 563 human-validated cells (0.7%) were used as training samples. The samples were evenly distributed among the ten types of landscape units present in the typology. We used 318 samples, independent from those provided for the classifier's training, to evaluate the classification outputs. A confusion matrix was used for the assessment. The assessment of classification models through the confusion matrix approach is a classical one with a consolidated tradition in evaluating model´s predictive performance [52]. This matrix summarizes the model's predictions against actual class labels, detailing the counts of true positive, true negative, false positive, and false negative classifications. It provides a set of metrics for accuracy, precision, recall, and F1-score, which enables a comprehensive understanding of the model's effectiveness.

Surveillance and control-oriented map for schistosomiasis: reclassifying landscape units on the map

The complete map of potential landscapes delineated by the ten types, as outlined in the Typology, was classified. To increase ease of interpretability, a second map was produced to demonstrate the diversity in landscape suitability for three specific scenarios linked to the schistosomiasis at the basin: (i) landscapes with the potential for ongoing transmission, (ii) landscapes deemed receptive, due to their current potential, to initiate or reintroduce active transmission, and (iii) landscapes resistant to the establishment of transmission, exhibiting comparatively lower potential within the current configuration of the basin to initiate or reintroduce active transmission. The set of reclassification rules that was established is depicted in Fig 5.

Fig 5. Reclassification system applied to the schistosomiasis risk landscape map obtained with supervised classification based on machine learning trained on the typology defined for the basin region.

These new landscape classes are described as follows:

- Potentially Active Landscape for Schistosomiasis: These are landscapes with water collections with habitats favourable to the intermediate host snail, in a transition area between the urban and peri-urban environment, with land use for horticulture, subject to flooding, with the possibility of untreated human waste being disposed of in the collection, and human presence that can develop the

infection. Corresponds to types 1 and 2 of the typology.

- Potentially Receptive Landscape to Schistosomiasis: These are landscapes with water collections with favourable habitats for intermediate host snails, in a transition area between urban and peri-urban environments, but also in rural areas, with water collections with riparian vegetation, and without or with low possibility of disposal of untreated human waste. And with low densities of human presence. Corresponds to types 3, 4, 5, 6 and 7 of the typology.

- Potentially Refractory Landscape to Schistosomiasis: These are landscapes with little potential for human transmission of schistosomiasis, either due to the absence of water collections or the absence of human presence. Corresponds to types 8, 9 and 10 of the typology.

Landscape metrics applied to the MP river basin

With the reclassified landscape map, a landscape-level analysis at the MP basin scale can be conducted. The context in which these landscapes exist and their connectivity with neighbouring landscapes can influence the potential risk of infection, the potential for disease reintroduction, and the effectiveness of control and elimination measures for this disease. To assess the spatial arrangement and connectivity of the established landscape categories on the reclassified map, a landscape ecology approach was employed [53]. The three landscape classes (in short) —active, receptive, and refractory— were vectorized and transformed into polygons, maintaining the total area of the MP basin as the scope for analysis. A set of landscape metrics was derived using Fragstats software [54]. The selected metrics for analyzing the spatial arrangement and connectivity of the polygons associated with the landscapes three classes primarily consist of aggregation metrics, measuring the tendency of patch types to be spatially aggregated. These metrics were used for the individualized analysis of each polygon within each class at two levels: (i) at the class level (C) and (ii) at the landscape level (L) for a comprehensive analysis of the three classes. The selected metrics for this analysis are listed and described in S1 Appendix - Landscape Metrics Description. We utilized the description, when necessary adjusted, from the Fragstats documentation [54].

Results

First, we present a summary of several outcomes regarding the construction phase of the variables associated with each landscape unit represented by the grid cells covering the entire MP basin. These variables were employed in the supervised classification process based on boost decision trees.

The methodology employed to rectify property overlaps in the CAR corrected 32,706 hectares of overlaps across 23 municipalities within the MP basin, as outlined in S1 Table - CAR Corrected Table. Among these municipalities, Iaras experienced the most significant correction in rural property areas. In this region, the variance between the area declared in the CAR and the corrected property areas amounted to 33.7%.

The outcome of the methodological strategies applied to identify the extent of wetland areas within the MP basin is presented in S2 Fig - Wetlands in the MP Basin. It illustrates drainage and HAND as indicators of freshwater presence, an essential element within environments prone to mollusk fixation. The wetland areas identified, primarily located surrounding the drainage network, cover 43.5% of the entire basin's area.

The schistosomiasis landscape risk map and its assessment

A boost decision tree-based supervised classification was applied to assign a specific landscape-unit type to individual grid cells based on their similarity to the provided training samples. The resulting classified map is depicted in Fig 6. Analysis of the map reveals a concentration of Types 1 and 2, more favourable to *S. mansoni* infected snails habitats, in close proximity to watercourses identified as sources of untreated sewage discharge. Type 5, representing agricultural areas near clean water bodies, was the most prevalent type, covering 31.8% of the study area. Types 1 and 2, posing higher potential for the presence of snails potentially infected with *S. mansoni*, accounted for 15.7%. Fig 7 provides a summary of the area coverage within the basin for each type identified in the typology of landscape units associated with schistosomiasis.

Fig 6. Schistosomiasis landscape risk map. (A) Mapping depicting the 10 types of landscape units within the MP basin utilizing the proposed typology, derived through supervised classification employing the boost decision tree method. (B) Emphasized section showcasing images associated with the municipality of Ourinhos.

Fig 7. Percentage of area within the basin for each landscape-unit type.

Classification accuracy assessment

A confusion matrix (Fig 8) was generated to assess the classification accuracy. Each evaluation sample's assigned value after classification was compared to the value provided by the analyst. The overall classification accuracy stood at 0.925, indicating that 92.5% of the 318 cells designated for evaluation were correctly classified.

Fig 8. Confusion matrix depicting the assessment of classification accuracy.

The assessment also involved a database containing snail data gathered from 2015 to 2018 in five municipalities within the Middle Paranapanema watershed [30]. Sampling encompassed 654 specific locations across 114 water bodies in the municipalities of Ourinhos, Ipaussu, Ribeirão do Sul, Assis, and Chavantes. Within these sampling points, 3151 snails belonging to at least one of the three intermediate host species of schistosomiasis were identified at 43 locations. Fig 9 displays a map featuring the classified landscape types alongside the collected *Biomphalaria* snails in the field. The assessment factored in the quantity of mollusks and the locations of collection points, each recording at least one individual from the species of intermediate host for schistosomiasis.

Fig 9. Map of classified landscape types and the collected *Biomphalaria* snails in the field.

The majority of the snails, constituting 74.6% of the total count, were collected in areas classified as Types 1 and 2. *B. glabrata* stands as the most susceptible neotropical snail host to the parasitic organism *S. mansoni* within the MP basin region. This particular snail species has historically been associated with the incidences of schistosomiasis among the human population living in the basin. Taking into account eight collection points that exclusively exhibited *B. glabrata*, the distribution was as follows: Type 1 with 1 collection point comprising 100 snails; Type 2 with 2 collection

points totaling 258 snails; Type 6 with 1 collection point accounting for 46 snails; Type 7 with 3 collection points amounting to 86 snails and a Type 9 landscape-unit pattern with 1 collection point with 12 snails. Of the 502 *B. glabrata* collected, 71.3% were found in landscape units classified as having a high potential for contact between hosts, potentially infected, and humans - specifically, Types 1 and 2. Additionally, 26.3% of the snails were located in landscape units identified as having a high potential for snail presence, albeit with a lower probability of encountering infected snails. Moreover, 2.4% of the snails, found solely at one collection point, were situated in a landscape unit classified as highly unsuitable for snail presence. In S3 Fig - *B. glabrata* in Landscape unit Type 9, we present some potential factors associated with this classification error.

Surveillance and control-oriented schistosomiasis landscape risk map

Fig 10 displays the reclassified map of the 10 landscape units identified through the supervised classification process, condensed into just three reclassified landscape units. This reorganization of landscape units provides a cartographic simplification intended for use by schistosomiasis surveillance and control services in the basin region. Three major landscapes of significance for surveillance and control actions at regional and local scales were established as: (i) Potentially Active Landscape, (ii) Potentially Receptive Landscape , and (iii) Potentially Refractory Landscape. In short, active, receptive and refractory landscapes for shistosomiasis in the basin.

Fig 10. Map depicting landscapes posing schistosomiasis risks, featuring three specific classes - active, receptive and refractory - for formulating monitoring and controlling strategies aimed at regional and local programs. Municipalities that reported autochthonous cases of schistosomiasis from 2006 to 2022 were highlighted.

A panel for communicating the results to the surveillance and control teams in the basin area

An online open-access interactive dashboard has been developed for the schistosomiasis surveillance and control teams in the basin area. The panel is currently in its preliminary version and is under discussion with both management and field teams. The current version of the panel presents maps resulting from the complete classification of the 10 types of landscapes and the reclassified map featuring potentially active, receptive, and refractory landscapes. The dashboard comprises four content pages: the first page displays the results from supervised classification of the 10 landscape types associated with schistosomiasis; the second page exhibits the reclassification outcomes with aggregated data forming landscape classes potentially active, receptive, or refractory to schistosomiasis; the third page accommodates the document outlining the typology of landscape patterns associated with schistosomiasis in the basin area and its description, while the fourth page shows the confusion matrix generated with evaluation samples. This version is available only in Portuguese as its objective is direct interaction with the services. This dashboard version can be accessed at (https://anrio0-vivian-silva.shinyapps.io/esquistossomose3/). For the construction of this Dashboard in its initial version, the R programming language and packages like Leaflet, Plotly, and Shiny were utilized as the foundation for the code development, available at https://github.com/vafds/esquistossomose.git. This version is available only in Portuguese as its aim is direct engagement with the services (Fig 11).

Fig 11. Second content page of the Schistosomiasis Dashboard. It shows the complete map of potentially active, receptive, and refractory landscapes in the interactive panel (Dashboard). The municipality being observed is Águas de Santa Bárbara. At the top, links to the other three content pages are seen.

Landscape metrics were examined at two levels on a basin scale: class (C) and landscape (L). At this broader scale the analysis was carried out using mainly aggregation measures for the schistosomiasis landscape risk classes. Table 3 shows these metrics results that are fully analysed in the discussion session.

Table 3. Metrics extracted from the reclassified map showing the three aggregated landscape types in the Middle Paranapanema river basin: Potentially active, receptive and refractory landscapes patterns. At the class level (C): Aggregation Index (AI), Proportion of Landscape (PLAND), Percentage of Like Adjacencies (PLADJ) and a Fragmentation Index (SPLIT) and at the landscape level (L): Mean Euclidean Nearest Neighbor Distance (ENN_MN).

**(L) Landscape Level**			
ENN_MN	688		
**(L) Landscape Level**	**(L) Potentially Active**	**(L) Potentially Receptive**	**(L) Potentially Refractory**
PLAND	38.1	51.8	10.1
AI	95.8	96.6	94.7
PLADJ	95.7	96.5	94.6
SPLIT	122.4	74.8	7194.0

Discussion

Schistosomiasis affects populations within their areas of life and experiences where, generally, the implementation of Water, Sanitation, and Hygiene (WASH) strategies is either non-existent or inadequately covered. This circumstance promotes varying degrees of contact and exposure between *S. mansoni*-infected intermediate hosts and humans. The landscapes shared by snails, the parasitic trematode, and humans are increasingly interwoven. These landscapes remain in constant transformation, with dynamics varying in speed. Changes in the physical landscape, alterations in native coverages, and modifications in land use, stemming from decisions associated with urban development models and ways of life imposed by societal choices, further drive these transformations. Associated with these socio-environmental dynamics are new dynamics resulting from the local implications of global-scale climate changes, such as an increase in the frequency and intensity of extreme phenomena like precipitation volume in the basin. These new elements add to the existing ones, shaping and reshaping the territories inhabited by humans and snails, thereby creating an integrated landscape, a physical-social complex that exhibits spatial and temporal arrangements leading to, or potentially leading to, the emergence, re-emergence, consolidation, or elimination of schistosomiasis. In summary, human collective actions, further compounded by the new situations arising from climate change reflected in local territories, can disrupt the local/regional landscape configurations and affect the establishment, maintenance and or elimination of the schistosomiasis transmission cycle at the basin scale.

This study aimed to examine this scale, that of the transformed landscape, as a pathogenic complex of schistosomiasis in the MP basin region. It highlights for the services the landscapes within a temporal scope, establishing the methodological

foundations for monitoring their transformation. In this sense, the methodology proposed and tested in this study for the MP basin establishes a new technical-instrumental possibility for the services associated with the surveillance and control of schistosomiasis in the area.

Taking these issues into consideration, the decision to construct a set of variables incorporating local data and knowledge to define the landscape types associated with schistosomiasis in the basin proved to be essential. The inclusion of a simplified model for detecting polluted water among the variables in this study was crucial for a classification outcome more aligned with the reality of domestic effluent discharge in the study area. Concerning the drainage network incorporated in the classification, adjustments regarding the relief forms aimed to prevent underestimation or overestimation of the hydrological configuration, avoiding major issues with the pollution model of specific sections within the network. The 12.5-meter spatial resolution of the PALSAR sensor aboard the ALOS satellite allowed for better recognition of terrain relief nuances compared to other freely used Digital Terrain Models (DTMs) commonly employed, such as the Shuttle Radar Topography Mission (SRTM), which possess lower spatial resolution [37, 55].

The HAND from the drainage network adjusted to the terrain relief was utilized to establish potential infection areas within the aquatic environment. Unlike the area of influence or buffer, which would indicate the horizontal distance between the drainage and a specific point, HAND accounts for variations in terrain altitude and includes only areas susceptible to flooding by adjacent watercourses. The cutoff point for the HAND metric disregarded areas with values exceeding the maximum height found through overlaying data on intermediate host snail collection in the region. This extrapolated data, applicable to the entire study area, is available for five municipalities within the Middle Paranapanema basin, leading to a non-individualized cutoff point for each municipality.

The correction of overlaps in Rural Environmental Registry (CAR) properties revealed that 2.83% of the total area of rural properties in the Middle Paranapanema basin exhibited rectified overlaps across 23 municipalities. Predominantly rural or peri-urban areas cover the major portion of the study area, despite most census tracts in the Middle Paranapanema basin being officially classified as ”urban”. This classification arises from the fact that the average size of rural sectors is larger than that of other classes, characterized by low household density.

An important limitation emerged in the land use and land cover map derived from satellite images, using the MapBiomas product. Because it is based on Landsat satellite data (with a 30-meter spatial resolution), the agricultural-related classes cannot discern more specific horticultural areas, such as vegetable and greens production belts, or even small backyard gardens in peri-urban areas. However, it currently stands as the available open and accessible data. By associating it with CAR data, there was a refinement, although not as desired, of these agricultural and livestock production areas. Undoubtedly, mapping with finner spatial resolution will yield a land use-related variable better suited for the classification process.

And lastly, but not less importantly, the generated maps offer us the significant opportunity to analyse the landscape’s situation in the basin, considering the spatial distribution and connectivity conditions related to classes potentially active, receptive, and refractory to schistosomiasis in the basin area.

Using the selected landscape metrics, a simple measure of the association between spatial patterns of landscapes and the processes involved in the transmission cycle is the area proportion, PLAND(C). This index was calculated at 10%, 52%, and 38% for landscapes classified as active, receptive, and refractory, respectively. Landscape metrics estimated at the class level show that the polygons constituting these landscapes have a

high level of aggregation. Landscapes classified as potentially active, despite occupying a smaller area proportion in the basin (10%) compared to the other classes, exhibit high aggregation indices, approaching 100 (maximum aggregation value). This can be observed in the AI(C) and PLADJ(C) indices presented in Table 3. At the landscape level, the average Euclidean distance to the nearest polygon, ENN_MN (L), considering all classes, was estimated at 688 meters. This result suggests that the polygons representing different landscapes are relatively close to each other, potentially connecting based on changes in climatic, socio-environmental, and political scenarios that govern the basin territories.

The level of landscape fragmentation is an important element to complement the analysis. We consider that the more subdivided the polygon classes are within a landscape, the higher the degree of isolation of that landscape in relation to risk classes. Isolation, concerning the risk of disease transmission, generally serves as a protective factor. For the landscape fragmentation analysis, we used the SPLIT(C) metric, which assessed each of the 3 classes. As a result, we obtained the highest value (7,194) for the potentially active class, indicating a high level of fragmentation and a potential isolation of some polygons composing this category. Although most of the area in this class is aggregated in larger polygons, numerous small, non-contiguous polygons are noticeable close to these aggregates (Fig 10). In summary, the spatial characteristics analysed reveal that polygons representing potentially active landscapes are largely grouped but demonstrate a high level of fragmentation and numerous isolated small patches. This helps to explain, partially, the low endemicity we found in the basin region. In the connectivity analysis using the ENN_MN distance index, we observed that polygons from the 3 landscape types are close to each other, indicating that the connection of the active class with the others (receptive and refractory) due to the spatial characteristics highlighted in this analysis could be facilitated in case of changes in the landscape configuration at the basin scale. This could be influenced by a set of endogenous and exogenous factors altering the land use and land cover mosaics and coverage situations for WASH-related services, particularly crucially important, the local and regional surveillance and control services that were not included in this modelling. Improvements in WASH-related conditions from 2000 to 2010, coupled with institutional strengthening of surveillance and control during the same period, are additional components that contribute to maintaining the observed low endemicity situation.

Extreme weather events (such as increased rainfall in short periods leading to flooding) or changes in surveillance actions can modify landscapes and transmission conditions in areas, connecting refractory and receptive landscapes to active landscapes, thereby altering the current scenario of schistosomiasis transmission control in the basin.

Conclusion

This work offers an effective basis for an integrative view for schistosomiasis risk mapping at the landscape scale for conducting research and promoting the reproducibility of methodologies. Our selection of free, open and reliable data with extensive national coverage enables the presented methodology to be replicated in other basins. A crucial element, besides the data, is the participation of local surveillance teams and the involvement of these teams and regional experts in defining the schistosomiasis typologies and the strategies for field visits in order to refine it.

The resulting classified landscape-unit types were rearranged to produce a map that expresses the gradient of possibility of local transmission of schistosomiasis. Landscape metrics were extracted from the final cartographic product, and their interpretation helped to understand the current scenario of low endemicity of schistosomiasis in the study area. The findings suggest that the areas with the highest risk of establishing the

schistosomiasis transmission cycle are more isolated and disconnected in the Middle Paranapanema river basin.

Our study has introduced an innovative methodology that leverages open Earth Observation data, open geotechnologies and open machine learning based supervised classification methods, that allows for introducing different levels of local and regional knowledge into the flow of technical procedures. Our findings amplify and extend existing mapping approaches by integrating environmental, epidemiological and socio-economic data in search of elucidating the intricate interplay between human society and nature, which together have influence on the transmission cycle of schistosomiasis. However, the analysis presented here is not exhaustive. For example, the intra-urban aspect could be further explored through broader-scale mapping initiatives to depict the disparities within urban areas. The spatial insights derived from this study serve as a valuable resource for implementing interventions aimed at reducing the population requiring treatment for neglected tropical diseases, aligning with the objectives of the World Health Organization and national schistosomiasis control and elimination programs.

However, some limitations of our study need to be addressed. Initially, the geographic distribution of *B. glabrata*, *B. tenagophila*, and *B. straminea* in the MP is unequal and scattered. Actually, *B. glabrata* habitats have been restricted to a few sites over time, which contrasts with the distributions of both of the other two species. Furthermore, the three snail hosts used in this study were considered equally susceptible to *S.mansoni*. However, important investigations have clearly established intra- and interspecific differences in neotropical *Biomphalaria/S. mansoni* [56–59]. Since *B. glabrata* is the most susceptible neotropical host to *S. mansoni*, this species has historically been connected to human occurrences of schistosomiasis in the MP basin. But how can we explain the decrease in schistosomiasis in the MP considering the fact that *B. tenagophila* is still abundant in water collections? Murray and Daszak [60] propose two ecological theories to explain the dynamics of diseases: 1-disturbance and 2-pathogen pooling. So, landscape disturbance in the MP basin might has favoured colonization of a more diverse freshwater snail species, including *B. occidentalis* and *B. peregrina*. This recent pattern of freshwater colonization would, in some way, act as a buffer for local schistosomiasis transmission. Can schistosomiasis strongly reemerge in the MP basin region? Considering the climate changes and its impact on floods; the rapid regional urbanisation processes, which has not been accompanied by an expansion in the coverage and quality of services associated with WASH strategies; the weakening of disease surveillance and control institutions by the state of São Paulo, through the dissolution of SUCEN-Superintendência de Controle de Endemias (Superintendence of Endemic Disease Control, established on April 17, 1970) by Decree no. 66.664 on April 14th, 2022, which addresses the effective dissolution of SUCEN, and the MP extensive vulnerables areas for flooding, indicate that the risk for infection is still a hypothesis for this region.

Supporting information

S1 Fig. Typology of landscape patterns associated with schistosomiasis in the Middle Paranapanema watershed. The figure shows 10 types of landscape units associated with schistosomiasis in the MP basin. To describe and characterise these types, we used (i) an image from the CBERS-4A satellite (L4; 2023/02/03; orbit/point:206/142; Sensor WPM/PAN 2m + RGB 8m; composition: R3G2B1 + pansharpening); (ii) a selected sample of land use and land cover data from MapBiomas (2022); (iii) a general description of that landscape associated with the type; (iv) Description of the spatial patterns observed in the landscape with quantitative and

qualitative characterisation for the elements that make up that landscape; (v) a description of the relevance of that landscape in the composition of the local risk for schistosomiasis; and (vi) the data used to generate the variables/indexes that took part in the supervised classification process based on a machine learning approach.

S2 Fig. Wetlands in the MP basin A) The map shows the outcome of the methodological strategies applied to identify the extent of wetland areas within the MP basin. It illustrates drainage network and high above nearest drainage (HAND) as indicators of freshwater presence. The wetland areas identified, primarily located surrounding the drainage network, cover 43.5% of the entire basin's area. B) The satellite image shows the difference between the two drainage network in different areas. The ultimate drainage network was constituted by the amalgamation of these two components, with its configuration influenced by the topographical features.

S3 Fig. *B. glabrata* in landscape unit Type 9. The figure shows the two *B. glabrata* in landscape unit Type 9 collection points near water bodies in cells with other land use and land cover. The classification rules, exemplified by parameters like the proportion of cell area occupied by forest vegetation or urbanized features, served to designate these areas as distinct from other landscape types.

S1 Appendix. Landscape Metrics Description. Description of the metrics employed in the analysis of individual polygons belonging to the three distinct classes at two levels: (i) at the class level (C) and (ii) at the landscape level (L).

S1 Table. CAR Corrected Table. The table presents the 23 municipalities within the MP basin wherein the methodology applied for rectifying overlaps in the Cadastro Ambiental Rural (CAR) corrected 32,706 hectares of overlap. Each municipality is accompanied by information detailing the declared area in the CAR, the corrected area subsequent to rectification, and the corresponding percentage disparity between these two values.

Authors contributions

VAFS, MK, RT and AMVM conceived and validated the methodology of the study. RGSP and RT prepared the malacological and the epidemiological data. VAFS and RSA prepared the environmental and population databases. VAFS performed the supervised classification and generate the landscape risk maps. VAFS, RT, MISE and AMVM conceived the schistosomiasis typology types. The first draft was written by VAFS and AMVM. The manuscript was revised by all authors. All authors contributed to the assessment of the classification results. All authors contributed to the article and approved the submitted version.

Acknowledgments

The authors thank the technical support of the fieldwork team of the Center for Health Control of the São Paulo State Health Authority based at Ourinhos assisting in all fieldwork activities and facilitating access to the freshwater bodies and snails collection. The authors also thank the Ministry of Health of Brazil for the production and free access dissemination of data from the Information System for Notifiable Diseases (SINAN) and the Epidemiological Surveillance Information System (SIVEP) and to the Brazilian Institute of Geography and Statistics (IBGE) for providing as open access

data the sociodemographic information. This study is a contribution from the LiSS-Laboratory for investigation of Socioenvironmental Systems and from the MOceanS -Monitoring Oceans from Space Laboratory at INPE to the Open Network for Water-Related Diseases (ONWARD) funded by the UKRI-GCRF Network Award (EP/T003820/1) and to the Belmont Collaborative Forum for Climate, Environment and Health Project S&CC - Integrated risk mapping and targeted snail control to support schistosomiasis elimination in Brazil and Cote d'Ivoire under future climate change.

References

1. World Health Organization [Internet]. [place unknown]: World Health Organization; 2023 Feb 01. Schistosomiasis: Fact sheets; [cited 2023 Nov 30]; Available from: https://www.who.int/news-room/fact-sheets/detail/schistosomiasis
2. Katz N. Inquérito Nacional de Prevalência da Esquistossomose mansoni e Geo-helmintoses. Belo Horizonte: CPqRR; 2018. 76 p.
3. Andrade AZ. A esquistossomose no Brasil após quase um século de pesquisas. Revista da Sociedade Brasileira de Medicina Tropical. 2002;35(5):509-513.
4. Coura J, Amaral R. Epidemiological and control aspects of schistosomiasis in Brazilian endemic areas. Memórias Do Instituto Oswaldo Cruz. 2004;99(suppl 1):13–19.
5. World Health Organization/UNICEF. Progress on household drinking water, sanitation and hygiene 2000–2022: special focus on gender. New York: World Health Organization (WHO) and United Nations Children's Fund (UNICEF), 2023.
6. Poague KIM, Mingoti IAH, Heller L. Water, sanitation and schistosomiasis mansoni: A study based on the Brazilian National Prevalence Survey (2011-2015). Ciência & Saúde Coletiva. 2023;28(2):363-372.
7. Silva LK, Barbosa LM, Kovach JD, Teixeira RS, Soares ES, Cardoso CW, Marques RVM, Santos TP, Reis MG, Blanton RE. The changing profile of schistosomiasis in a changing urban landscape. Int J Parasitol. 2020;50(1):27-34.
8. Gomes ECS, Silva IEP, Nascimento WRC do, Loyo RM, Domingues ALC, Barbosa CS. Urban schistosomiasis: An ecological study describing a new challenge to the control of this neglected tropical disease. The Lancet Regional Health - Americas [Internet]. 2022 Apr 1;8:100144. Available from: https://www.sciencedirect.com/science/article/pii/S2667193X2100140X
9. Klohe K, Koudou BG, Fenwick A, Fleming F, Garba A, Gouvras A, et al. A systematic literature review of schistosomiasis in urban and peri-urban settings. PLOS Neglected Tropical Diseases. 2021 Feb 25;15(2):e0008995.
10. Chaves CF, Sabino-Santos G, Cedraz FMA, Santos-Muccillo P, Filho JR, Zanardi VS, et al. Evidence for local transmission and maintenance of schistosomiasis in an urban neighborhood in Northeast Brazil. Transboundary and Emerging Diseases. 2022;69:3153–3159.
11. Santos MCS, Oliveira GLd, Mingoti SA, Heller L. Effect of environmental factors in reducing the prevalence of schistosomiasis in schoolchildren: An analysis of three extensive national prevalence surveys in Brazil (1950–2018). PLOS Neglected Tropical Diseases. 2023 Jul 17;17(7):e0010804.

12. Palasio RG, Xavier IG, Chiaravalotti-Neto F, Tuan R. Diversity of Biomphalaria spp. freshwater snails and associated mollusks in areas with schistosomiasis risk, using molecular and spatial analysis tools. Biota Neotropica. 2019;19(4).

13. Dias LC, Ueta MT, Guaraldo AM. Suscetibilidade de Biomphalaria glabrata, B. straminea e B. tenagophila a diferentes cepas de Schistosoma mansoni. Revista do Instituto de Medicina Tropical de São Paulo. 1987 Jul-Aug;29(4):205-12.

14. McCreesh N, Arinaitwe M, Arineitwe W, et al. Effect of water temperature and population density on the population dynamics of Schistosoma mansoni intermediate host snails. Parasites & Vectors. 2014;7(1):503.

15. McCreesh N, Booth M. Challenges in predicting the effects of climate change on Schistosoma mansoni and Schistosoma haematobium transmission potential. Trends in Parasitology. 2013;29(11):548–555.

16. Leo GAD, Stensgaard AS, Sokolow SH, N'Goran EK, Chamberlin AJ, Yang GJ, et al. Schistosomiasis and climate change. BMJ. 2020; 371 :m4324.

17. Sokolow SH, Jones IJ, Jocque M, et al. Nearly 400 million people are at higher risk of schistosomiasis because dams block the migration of snail-eating river prawns. Philosophical Transactions of the Royal Society B: Biological Sciences. 2017 Jun 5;372(1722):20160127.

18. Reitzug F, Ledien J, Chami GF. Associations of water contact frequency, duration, and activities with schistosome infection risk: A systematic review and meta-analysis. PLOS Neglected Tropical Diseases. 2023 Jun 14;17(6):e0011377

19. Habib MR, Lv S, Rollinson D, Zhou X-N. Invasion and Dispersal of Biomphalaria Species: Increased Vigilance Needed to Prevent the Introduction and Spread of Schistosomiasis. Frontiers in Medicine. 2021, 8:614797.

20. World Health Organization. Ending the neglect to attain the Sustainable Development Goals: a road map for neglected tropical diseases 2021–2030. Geneve: World Health Organization; 2020.

21. World Health Organization. WHO guideline on control and elimination of human schistosomiasis. Geneva: World Health Organization; 2022

22. Wiegand RE, Fleming FM, de Vlas SJ, Odiere MR, Kinung'hi S, King CH, et al. Defining elimination as a public health problem for schistosomiasis control programmes: beyond prevalence of heavy-intensity infections. Lancet Global Health. 2022;10(9):e1355–1359.

23. Reinhard-Rupp J, Klohe K. Developing a comprehensive response for treatment of children under 6 years of age with schistosomiasis: research and development of a pediatric formulation of praziquantel. IInfectious Diseases of Poverty. 2017 Aug 3;6(1).

24. Ziegler M. Landscapes of Disease. Landscapes. 2016;17(2):99-107.

25. Lambin EF, Tran A, Vanwambeke SO, Linard C, Soti V. Pathogenic landscapes: Interactions between land, people, disease vectors, and their animal hosts. International Journal of Health Geographics. 2010;9(1):54.

26. McManus DP, Dunne DW, Sacko M, Utzinger J, Vennervald BJ, Zhou X. Schistosomiasis. Nature Reviews Disease Primers. 2018;4(1):13.

27. Ferreira JM, Meira JA. Três casos de esquistossomose mansoni procedentes do interior do estado de São Paulo (Ourinhos, Palmital e Ipauçu). Foco autóctone na cidade de Ourinhos. Revista Paulista de Medicina . 1952;41(1):15-8.

28. Vaz JF, et al. newblockLevantamento planorbídico do Estado de São Paulo (Brasil): 4a Região Administrativa. Revista De Saude Publica. 1987;21(5):371-379.

29. SINAN-MS. Transferência de Arquivos – DATASUS [Internet]. Available from: https://datasus.saude.gov.br/transferencia-de-arquivos

30. Palasio RGS, Bortoleto AN, Rosa-Xavier IG, Andrighetti MTM, Tuan R, Chiaravalloti-Neto F. Schistosomiasis in the Middle Paranapanema river region, state of São Paulo, Brazil: Does it matter today for public health? Revista da Sociedade Brasileira de Medicina Tropical. 2019;52::e20180447.

31. Eduardo MBP. Avaliação da Esquistossomose no Estado de São Paulo. Secretaria do Estado da Saúde, Coordenadoria de Controle de Doenças, Centro de Vigilância Epidemiológica, Divisão de Doenças de Transmissão Hídrica e Alimenta. 2008.

32. Instituto Brasileiro de Geografia e Estatística. Relação da População para publicação no DOU em 2023 - Municípios. IBGE. 2023.

33. Palasio RG, Xavier IG, Chiaravalloti-Neto F, Tuan R. Diversity of Biomphalaria spp. freshwater snails and associated mollusks in areas with schistosomiasis risk, using molecular and spatial analysis tools. Biota Neotropica. 2019;19(4).

34. Souza CM, Shimbo ZJ, Rosa MR, Parente LL, Alencar AA, Rudorff BFT, et al. Reconstructing Three Decades of Land Use and Land Cover Changes in Brazilian Biomes with Landsat Archive and Earth Engine. Remote Sensing. 2020;12(17):2735.

35. OpenStreetMap. OSM History Dump [Internet]. OpenStreetMap Contributors. [cited 2023 Dec 15] Available online: https://planet.openstreetmap.org/planet/full-history

36. Mobity Typology Database. Mobility Typologies for the Metropolitan Region of the Paraíba Valley and North Coast (RMVPLN) Creators.Anazawa, Tathiane Mayumi; Monteiro, Antonio Miguel Vieira. ZENODO. [Internet]. Avaliable from: https://zenodo.org/records/7097182.

37. Alaska Satellite Facility. ALOS PALSAR Radiometric Terrain Corrected low res. Alaska Satellite Facility: University of Alaska Fairbanks, Fairbanks. 2015.

38. Rennó CD, Nobre AD, Cuartas LA, Soares JV, Hodnett MG, Tomasella J, et al. HAND, a new terrain descriptor using SRTM-DEM: Mapping terra-firme rainforest environments in Amazonia. Remote Sensing of Environment. 2008;112(9):3469-81.

39. Valeriano MM, Rossetti DF, Albuquerque PCG. Topodata: desenvolvimento da primeira versão do banco de dados geomorfométricos locais em cobertura nacional. In: Simpósio Brasileiro de Sensoriamento Remoto [Internet]. São José dos Campos, Brazil: INPE; 2009. p. 1-8. Available from: http://www.dsr.inpe.br/topodata/acesso.php

40. Abreu ES, Rosim S, Renno CD, Oliveira JRF, et al. Terrahidro — A Distributed Hydrological System to delimit large basins. In: 2012 IEEE International Geoscience and Remote Sensing Symposium. Munich, Germany; 22-27 July 2012. p. 546-549.

41. Instituto Brasileiro de Geografia e Estatística. Censo Demográfico 2010 [Internet]. [cited 2023 Oct 22]. Available from: https://www.ibge.gov.br/en/statistics/multi-domain/culture-recreation-and-sports/18391-2010-population-census.html.

42. Instituto Brasileiro de Geografia e Estatística. Censo Demográfico 2022 - Malha de Setores Censitários [Internet]. [cited 2023 Jun 12]. Available from: https://www.ibge.gov.br/en/geosciences/territorial-organization/territorial-meshes/2998-np-mesh-of-enumeration-areas/28114-malhas-de-setores-censitarios-divisoes-intramunicipais-2.html.

43. SICAR, Pub. L. No. 12.651/2012 (May 5, 2014).

44. Matias MR, Milare G, Escada MIS, Monteiro AMV. Q-CleanCAR: Um complemento QGIS para remoção guiada de sobreposições de polígonos de imóveis rurais do Cadastro Ambiental Rural (CAR). In: XXIII Brazilian Symposium on Geoinformatics. São José dos Campos, Brazil: INPE; 28-30 November 2022. p. 417-422. Available from: http://mtc-m16c.sid.inpe.br/col/sid.inpe.br/mtc-m16c/2022/12.16.17.19/doc/thisInformationItemHomePage.html.

45. Anjos, R. S. Relatório de Projeto Cartografia das Paisagens Patogênicas envolvidas na ocorrência e dinâmica da Esquistossomose na Microrregião de Ourinhos, SP. São José dos Campos: Instituto Nacional de Pesquisas Espaciais (INPE); 2023.

46. Carneiro TGS, Andrade PR, Câmara G, Monteiro AMV, Pereira RR. An extensible toolbox for modeling nature–society interactions. Environmental Modelling & Software. 2013;46:104–117.

47. start - TerraLib5 [Internet]. www.dpi.inpe.br. [cited 2023 Dec 17]. Available from: http://www.dpi.inpe.br/terralib5/wiki/doku.php?id=start

48. Quinlan JR. Introduction of decision trees. Machine Learning. 1986;1:81–106.

49. Freund Y, Schapire RE. Experiments with a new boosting algorithm. In: Thirteenth International Conference on Machine Learning. Bari, Italy: 3-6 July 1996. p. 148–156.

50. Quinlan JR. Data Mining Tools See5 and C5.0. 2004.

51. Körting, T.S., Fonseca, L.M.G., Câmara, G. GeoDMA - Geographic Data Mining Analyst. Computers & Geosciences [Internet]. 2013 Aug 1 [cited 2023 Dec 17];57:133–45. Available from: https://www.sciencedirect.com/science/article/pii/S0098300413000538

52. Goodman LA, Kruskal WH. Measures of Association for Cross Classifications. Journal of the American Statistical Association. 1954;49(268):732-764

53. Forman RTT. Land mosaics: the ecology of landscapes and regions. Cambridge University Press, Cambridge, England. 1995.

54. McGarigal K, Cushman SA, Ene E. FRAGSTATS v4: Spatial Pattern Analysis Program for Categorical Maps. Technical report; Department of Environmental Conservation University of Massachusetts: Amherst, MA, USA, 2002.

55. Farr TG, et al. The shuttle radar topography mission. Reviews of Geophysics. 2007;45:595–617.

56. Paraense WL, Correa LR. Variation in susceptibility of populations of Australorbis glabratus to a strain of Schistosoma mansoni. Revista do Instituto de Medicina Tropical de São Paulo. 1963 Jan-Feb;5:15-22.

57. Magalhães LA, Dias LC. Estudo da suscetibilidade da Biomphalaria glabrata de Ourinhos (SP), á infecção pelo Schistosoma mansoni de Belo Horizonte (MG), e de São José dos Campos (SP). Revista de Saúde Pública. 1973 Sep;7(3):295-7.

58. Dias LCS, Ueta MT, Guaraldo AMA. Suscetibilidade de biomphalaria glabrata, B. straminea e B. tenagophila a diferentes cepas de schistosoma mansoni. Revista do Instituto de Medicina Tropical de São Paulo [online]. [cited 2024 Jan 2]. 1987 Aug 1;29(4):205–12. Available from: https://doi.org/10.1590/S0036-46651987000400004.

59. Dias LCS, Glasser CM, Etzel A, et al. The epidemiology and control of schistosomiasis mansoni where Biomphalaria tenagophila is the snail host. Revista de Saúde Pública. 1988;22(5):462-463.

60. Murray KA, Daszak P. Human ecology in pathogenic landscapes: two hypotheses on how land use change drives viral emergence. Current Opinion in Virology [Internet]. 2013 Feb [cited 2023 Dec 2];3(1):79–83. Available from: https://www.ncbi.nlm.nih.gov/pmc/articles/PMC3713401

Figure

Click here to access/download;Figure;Fig1.tiff

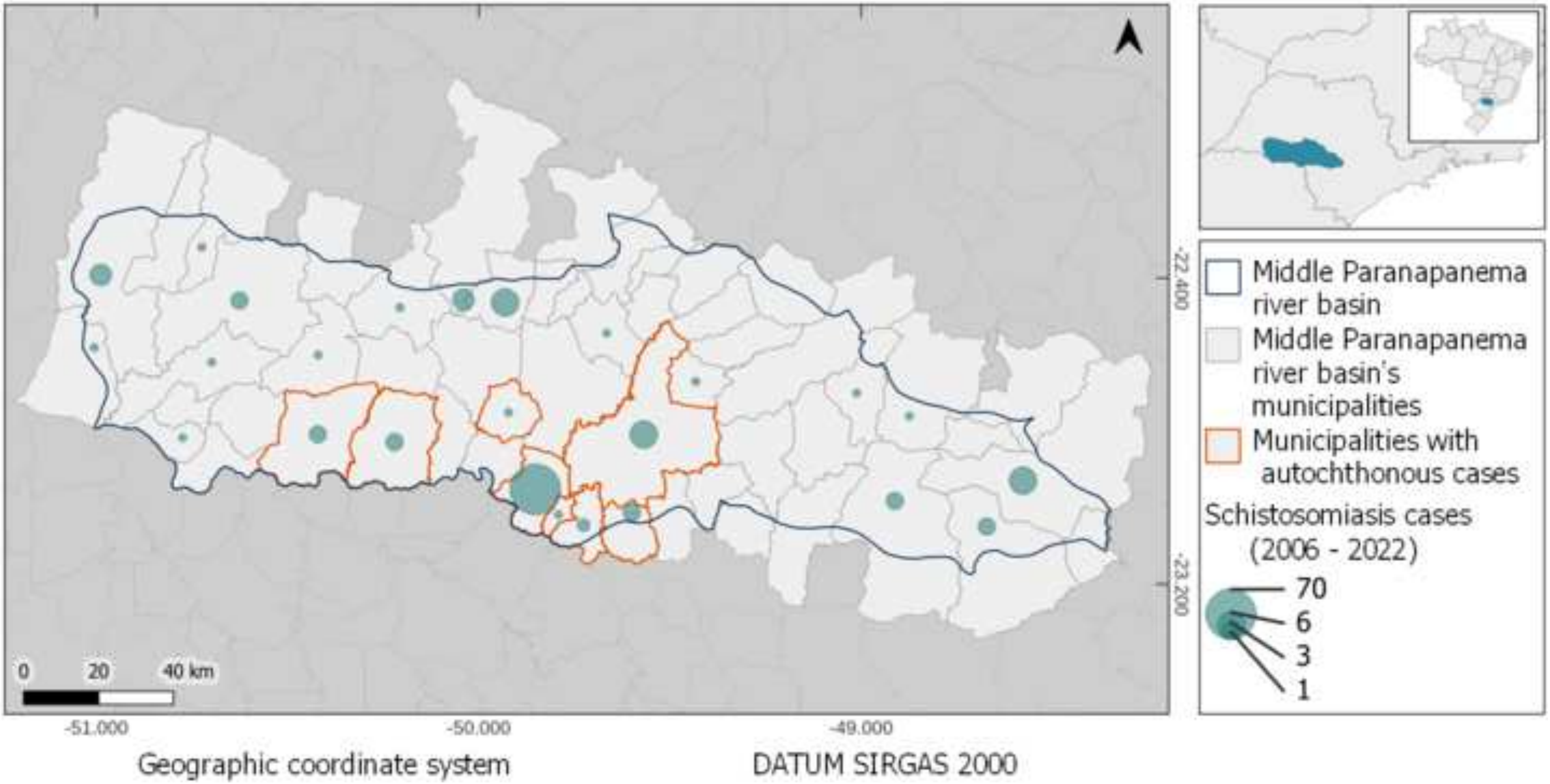

Click here to access/download;Figure;Fig2.tiff

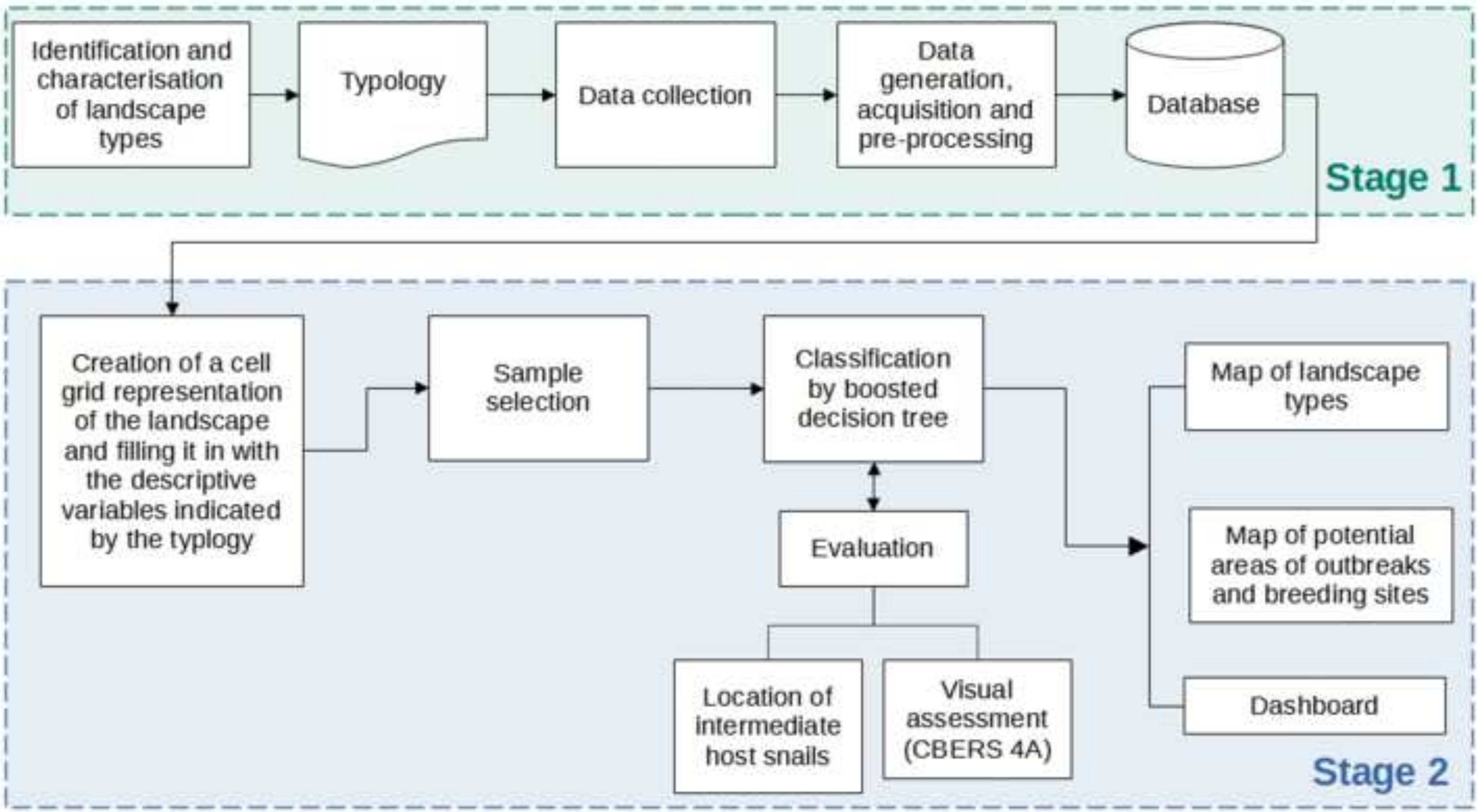

Figure

Click here to access/download;Figure;Fig3.tiff

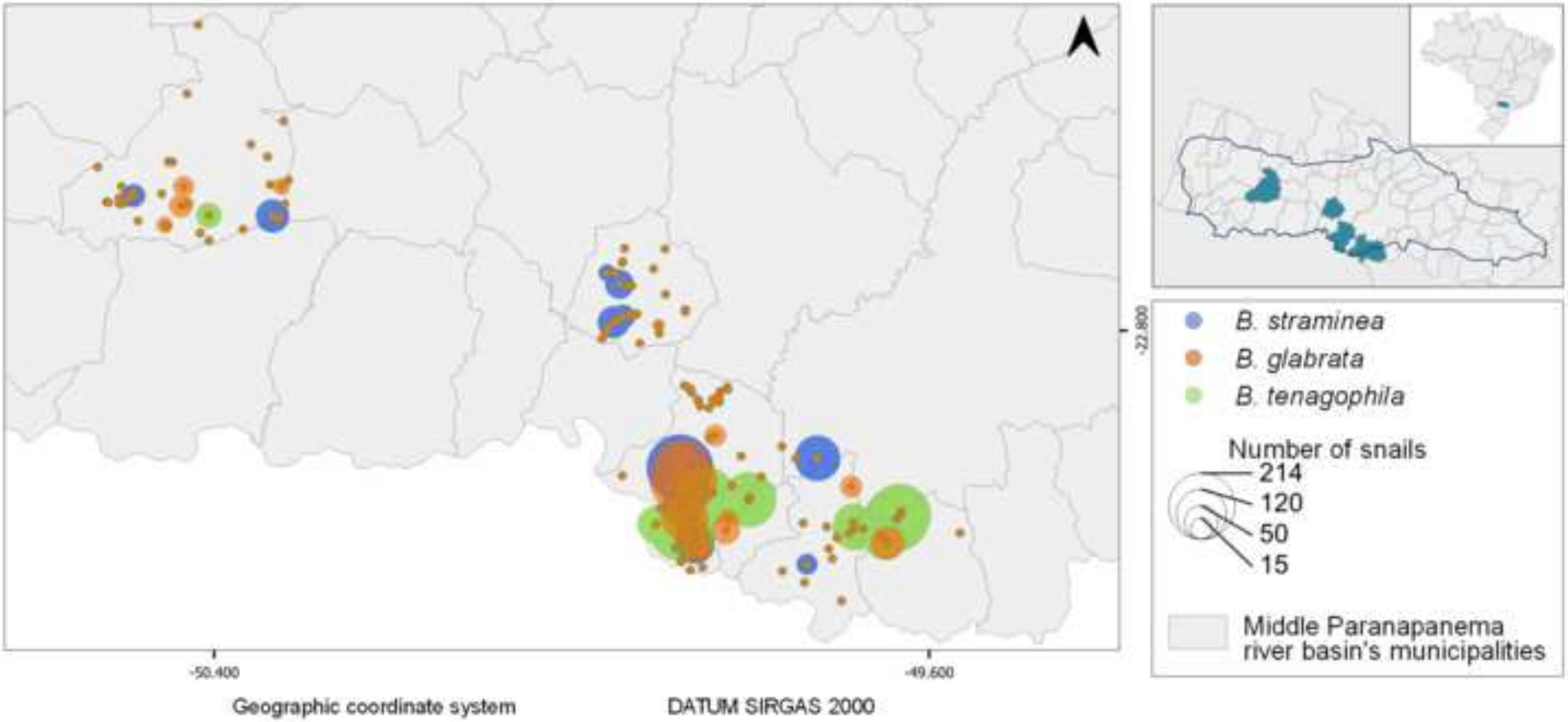

Figure

Click here to access/download;Figure;Fig4.tiff

Type identification	Photograph and image from CBERS-4A satellite (L4; 2023/02/03; orbit/point 206/142; Sensor: WPM/PAN 2m + RGB 8m; composition: R3G2B1 + pansharpening.	Sample of land use and land cover (LULC) data from MapBiomas (2022), spatial resolution 30m.	Description of the landscape.	Description of the spatial pattern with quantitative and qualitative specification of the elements that compose the type.	Relevance for the risk of local human schistosomiasis infection	Data used in type classification
1	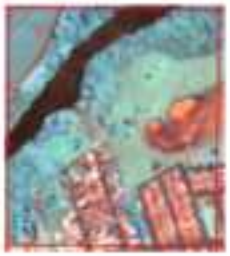	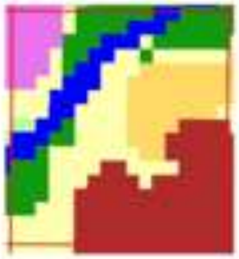	Small-scale agricultural areas near water bodies that receive untreated domestic wastewater discharge.	Cells containing small-scale agricultural infrastructure, containing surface water bodies or areas up to 29 m high above the nearest drainage with discharge of untreated domestic wastewater discharge.	Receptive area for schistosomiasis. Area that fulfils the conditions needed to maintain the parasite cycle.	- Land use and land cover (MapBiomas, 2022), 30 m of spatial resolution<br>- Relief-adjusted drainage network (PALSAR/ALOS, 2014)<br>- High above nearest drainage – HAND (PALSAR/ALOS, 2014)<br>- Micro-size farms and small rural properties of agricultural production (CAR, 2022), shapefile, UTM 22 S / SIRGAS 2000<br>- Census tract type (IBGE, 2022) census tract vetorial database, shapefile, geographic coordinates/ SIRGAS 2000<br>- Households presence (IBGE, 2010)<br>- Mobility Typology (Open Street Map, 2022)<br>- Polluted water bodies<br>- Slope of water bodie's edges

Figure

Click here to access/download;Figure;Fig5.tiff

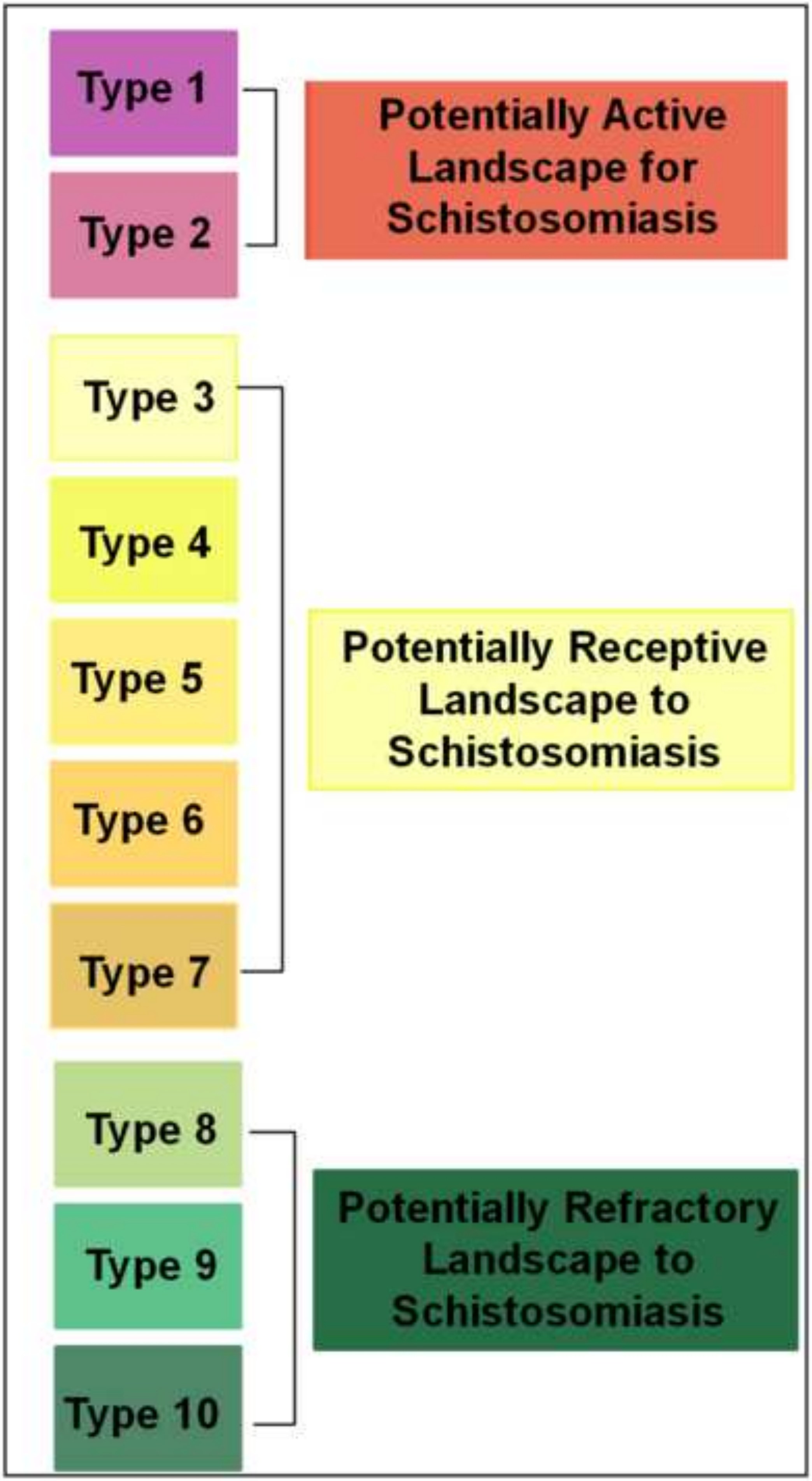

Figure
Click here to access/download;Figure;Fig6.tiff

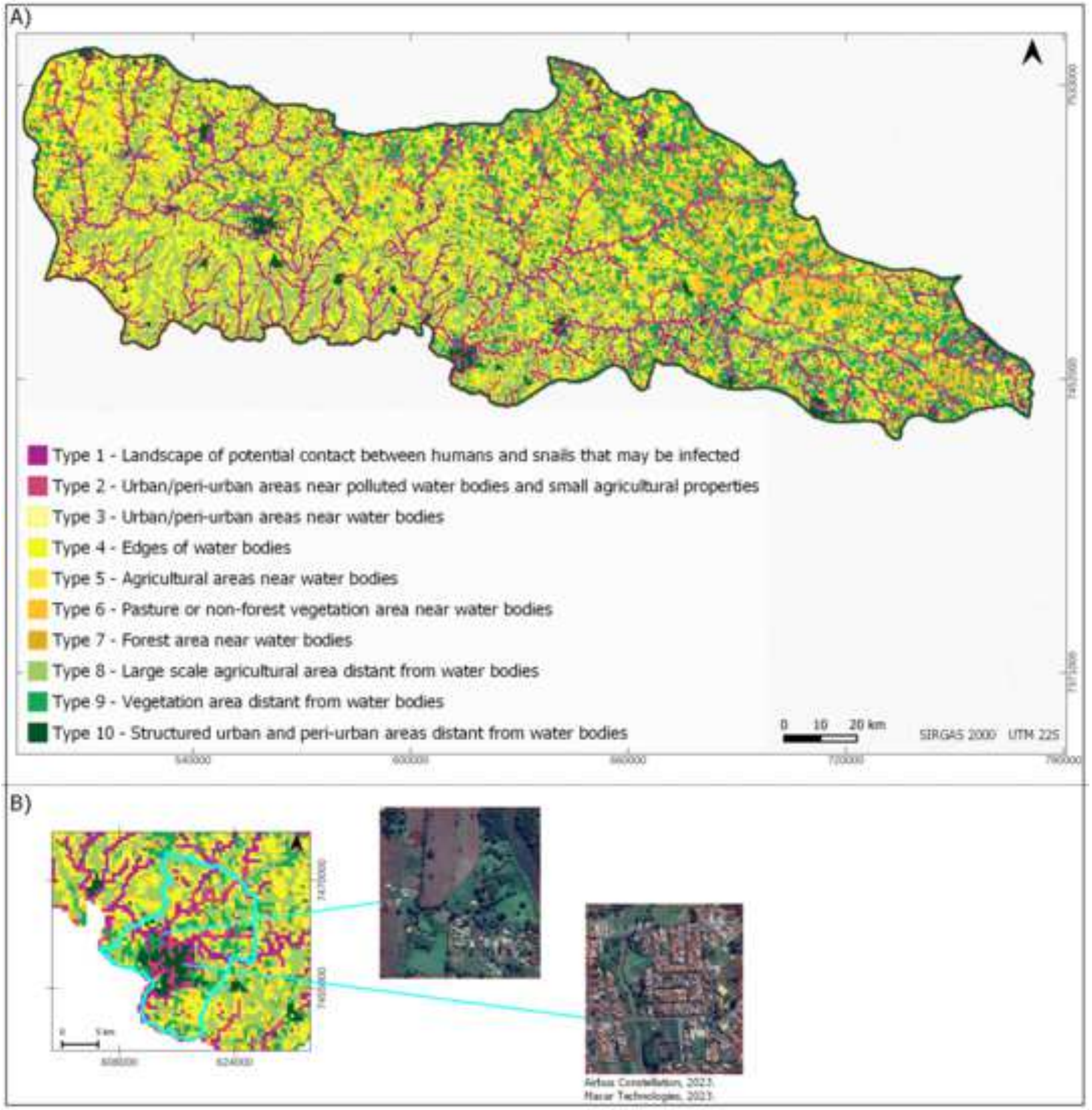

 Click here to access/download;Figure;Fig7.tiff

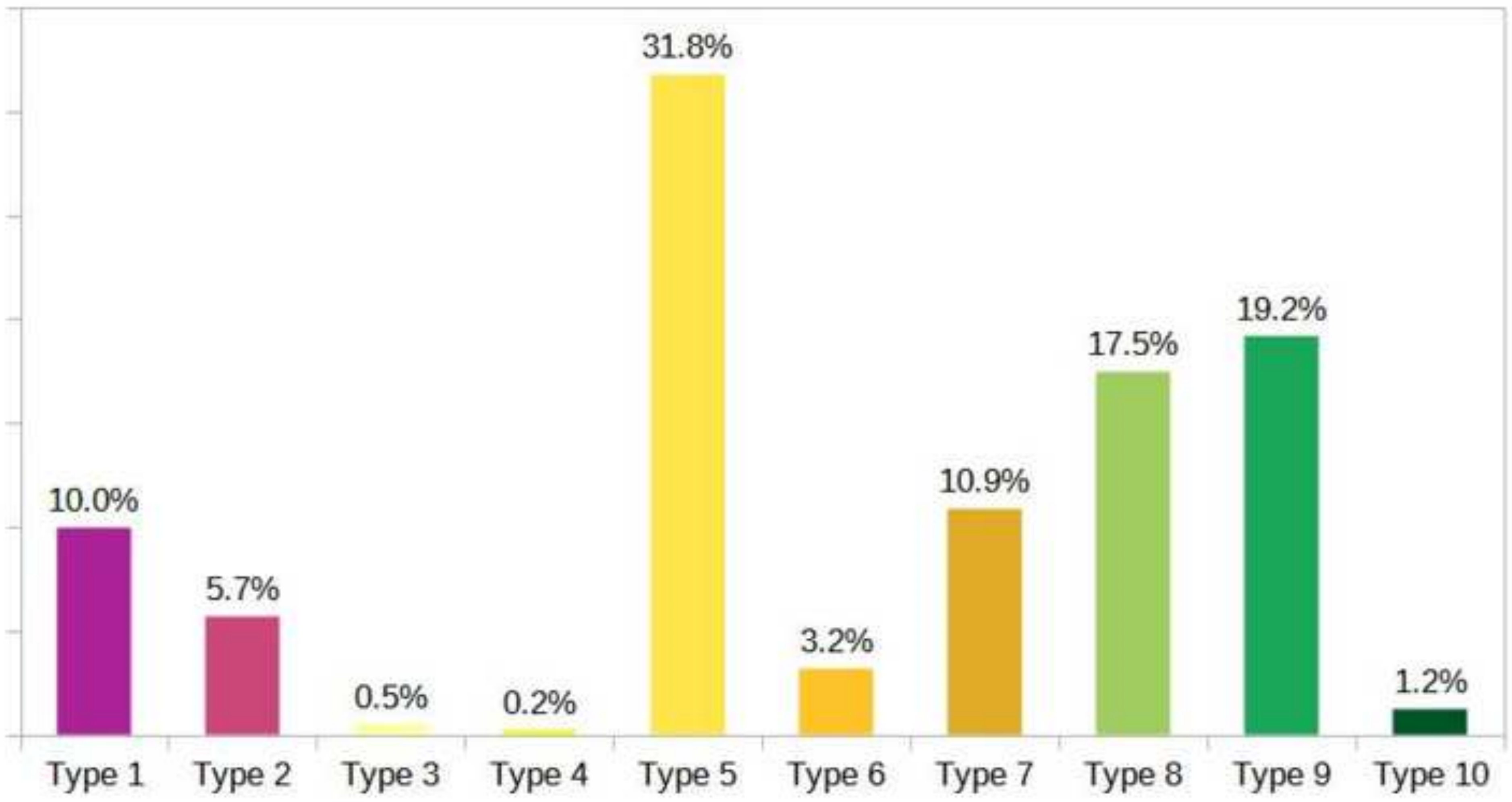

Figure

Click here to access/download;Figure;Fig8.tiff

		Classification												
		Type 1	Type 2	Type 3	Type 4	Type 5	Type 6	Type 7	Type 8	Type 9	Type 10	Total	User accuracy	F1 score
Real	Type 1	27										27	1.000	1.000
	Type 2		28									28	1.000	0.966
	Type 3			21	2							23	0.913	0.933
	Type 4				20							20	1.000	0.952
	Type 5					34	2		2			38	0.895	0.883
	Type 6			1		2	28					31	0.903	0.903
	Type 7						1	43		6		50	0.860	0.896
	Type 8					3			35			38	0.921	0.933
	Type 9		2					3		25		30	0.833	0.820
	Type 10										35	35	1.000	1.000
	Total	27	30	22	22	39	31	46	37	31	35	320		
	Producer accuracy	1.000	0.933	0.955	0.909	0.872	0.903	0.935	0.946	0.806	1.000			
	Global accuracy		0.925											

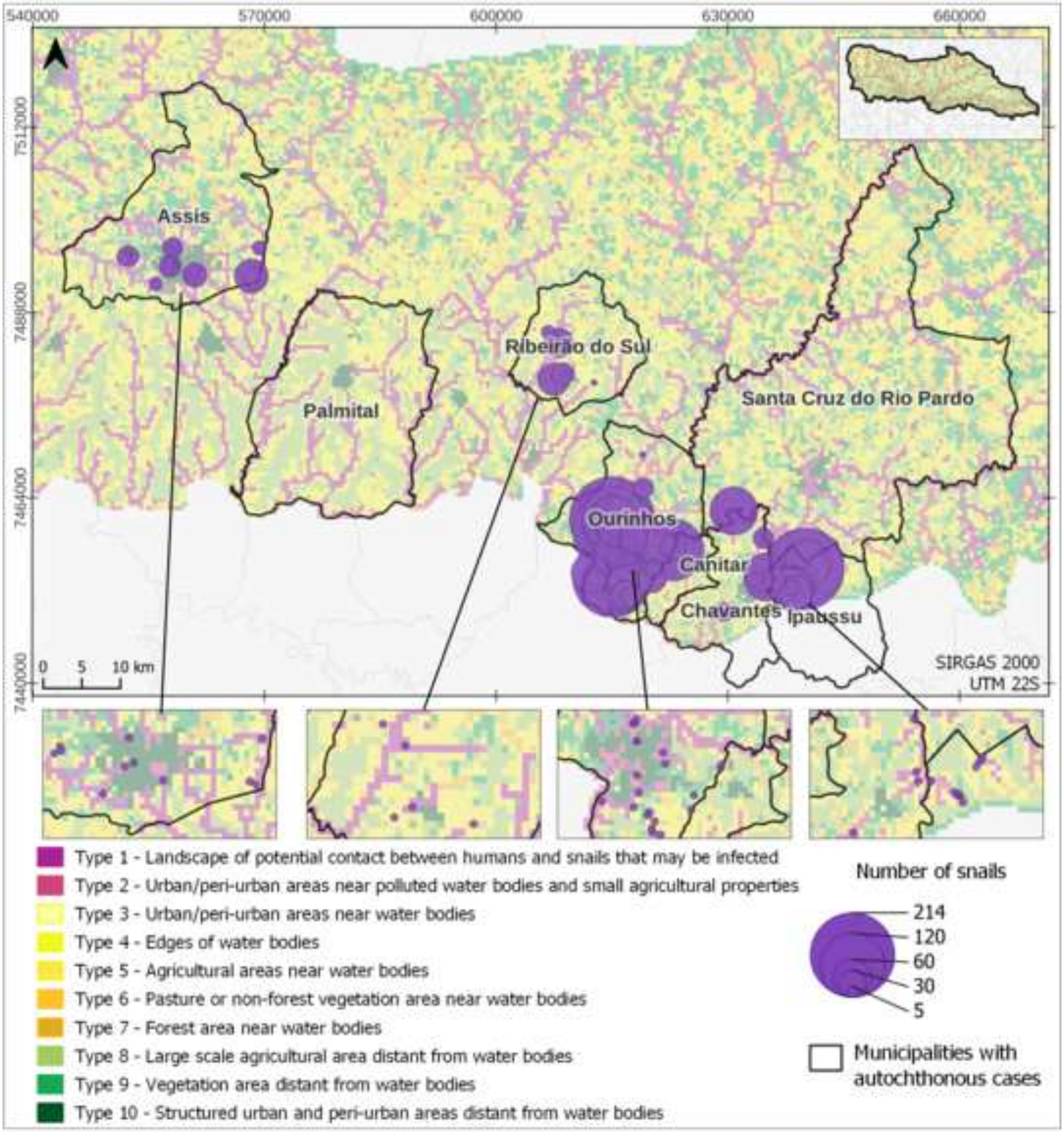
540000
570000
600000
630000
660000
7512000
7488000
7464000
7440000
Assis
Palmital
Ribeirão do Sul
Santa Cruz do Rio Pardo
Ourinhos
Canitar
Chavantes
Ipaussu
0 5 10 km
SIRGAS 2000
UTM 22S
Type 1 - Landscape of potential contact between humans and snails that may be infected
Type 2 - Urban/peri-urban areas near polluted water bodies and small agricultural properties
Type 3 - Urban/peri-urban areas near water bodies
Type 4 - Edges of water bodies
Type 5 - Agricultural areas near water bodies
Type 6 - Pasture or non-forest vegetation area near water bodies
Type 7 - Forest area near water bodies
Type 8 - Large scale agricultural area distant from water bodies
Type 9 - Vegetation area distant from water bodies
Type 10 - Structured urban and peri-urban areas distant from water bodies
Number of snails
214
120
60
30
5
Municipalities with autochthonous cases

Click here to access/download;Figure;Fig10.tiff

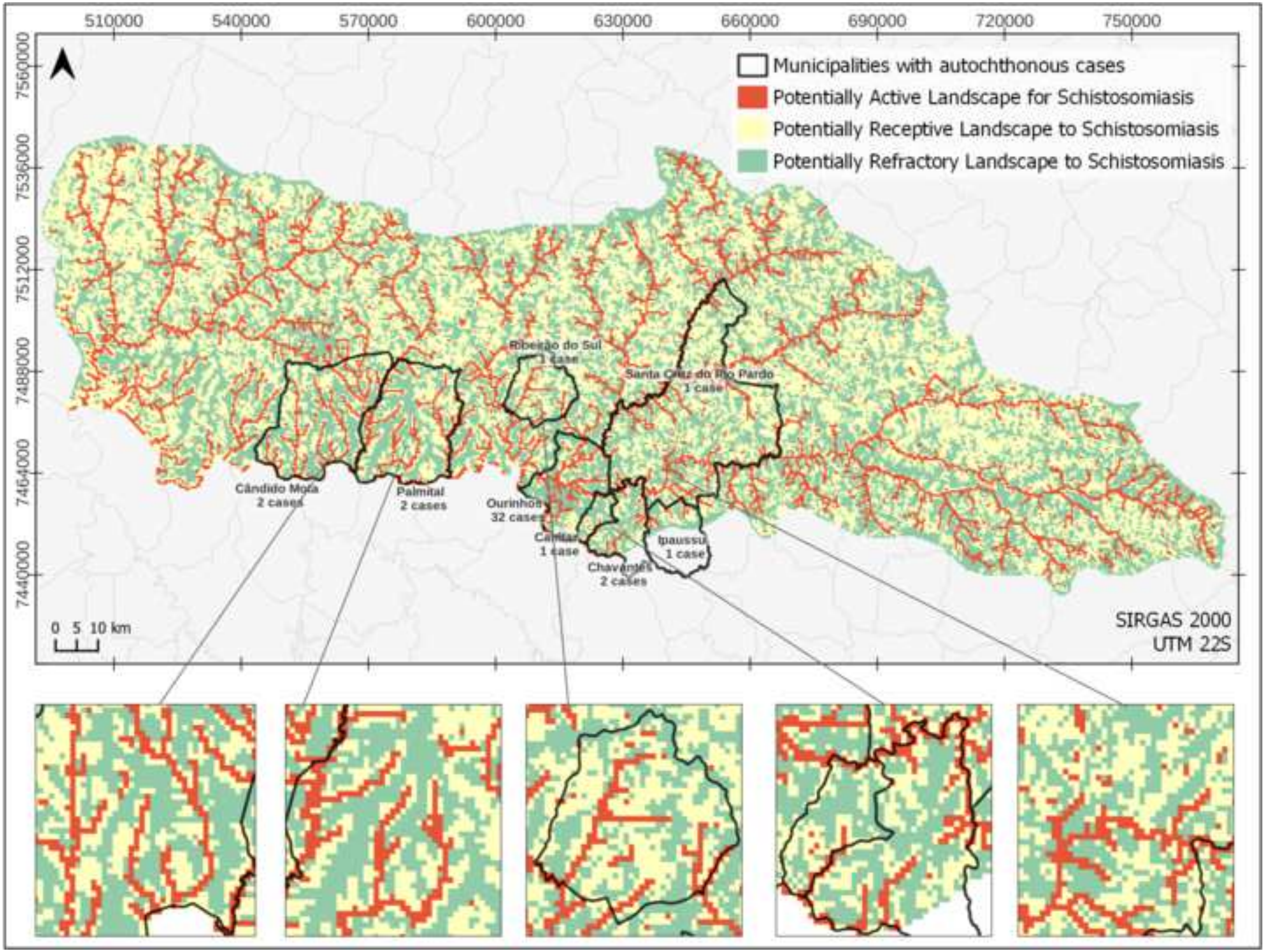

Figure Click here to access/download;Figure;Fig11.tiff

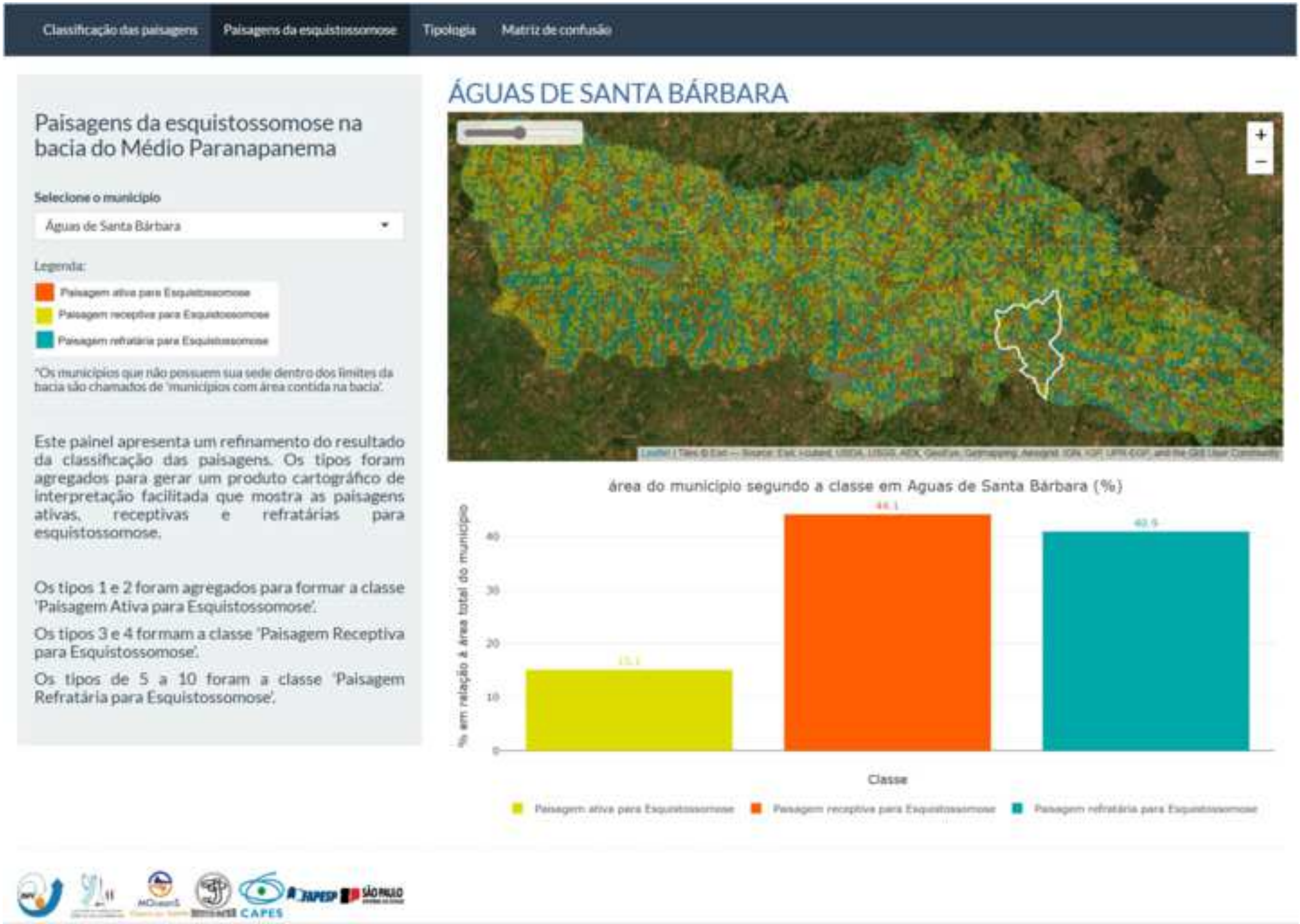

Supporting information figure

Click here to access/download
Supporting Information
S1 Fig.tiff

Supporting information figure

Click here to access/download
Supporting Information
S2 Fig.tiff

Supporting information figure

Click here to access/download
Supporting Information
S3 Fig.tiff

Supporting information appendix

Click here to access/download
Supporting Information
S1 Appendix.pdf

Supporting information table

Click here to access/download
Supporting Information
S1 Table.pdf
