# Supplementary material for: Mapping schistosomiasis risk landscapes and implications for disease control: A case study for low endemic areas in the Middle Paranapanema river basin, São Paulo, Brazil
Source: PLoS Negl Trop Dis. 2024 Nov 4;18(11):e0012582. doi: 10.1371/journal.pntd.0012582 (PMC11563476; doi:10.1371/journal.pntd.0012582)
Supplement: Attachment — Submitted filename: Response letter.pdf [file pntd.0012582.s009.pdf]

Dear Dr. Paul O. Mireji,

I am writing on behalf of all authors of our research paper to express our gratitude for the constructive feedback provided by the reviewers on our manuscript titled "**Mapping schistosomiasis risk landscapes and implications for disease control: a case study for low endemic areas in the Middle Paranapanema river basin, São Paulo, Brazil**" submitted to *PLOS Neglected Tropical Diseases*.

We have considered all the reviewers' comments and made every effort to respond to them. We have provided the necessary clarifications regarding the lack of ethical approval, as we did not use confidential data or data from individual patients or even specific cases of schistosomiasis in our analysis. We also worked on improving the writing of some parts of the text.

We have provided a point-by-point response below on how we have addressed the reviewers' feedback. Our response is highlighted in red.

We are hopeful and confident that the manuscript now meets the standards for publication in PLOS Neglected Tropical Diseases. We appreciate the time and effort the reviewers have put into reviewing our work, and I look forward to hearing from you soon.

Sincerely,

Vivian Alessandra Ferreira da Silva

Remote Sensing and Geoinformatics Graduate Program

National Institute for Space Research

Methods......2
Results......3
Conclusions......4
Editorial and Data Presentation Modifications......4
Summary and General Comments......7

Methods

Reviewer #1:

- Are the objectives of the study clearly articulated with a clear testable hypothesis stated? Yes
- Is the study design appropriate to address the stated objectives? Yes
- Is the population clearly described and appropriate for the hypothesis being tested? Yes
- Is the sample size sufficient to ensure adequate power to address the hypothesis being tested? Yes
- Were correct statistical analysis used to support conclusions? Yes
- Are there concerns about ethical or regulatory requirements being met? Yes

We appreciate your positive feedback.

Reviewer #2:

The research objectives are clearly defined, and the design seems appropriate for the goals. However, the excerpt does not provide enough information to fully assess the sample size's adequacy or ethical considerations. The statistical analysis appears to be correctly applied based on the information given.

Thank you for your comments. Regarding the lack of ethical considerations, we should clarify that we did not use confidential data or data from individual patients in our analysis.

We have changed the text regarding the sample size used in the landscape classification process to make it clearer:

Lines 324-328: "Of the 73,698 cells, we selected 881 cells (1.2%) evaluated by field work experts, with 563 (63%) used as training samples and 318 (37%) used as testing samples to assess the classification results. The samples were evenly distributed among the ten types of landscape units present in the typology and spatially distributed along the study area."

Reviewer #3:

The objectives of the study are clearly articulated.
The study design is appropriate to address the stated objectives.
The population is clearly stated.
The sample size is adequate.

The correct statistical analysis were used.
The authors should inculde an ethical statement for the study.

The PLOS NTD editorial Guideline states that an *Ethics statement* should be provided, “If the study made use of human or animal subjects and/or tissue, you must provide an ethics statement.

Our study proposes and uses a methodological approach that integrates open remote sensing, environmental, socio-economic and epidemiological data and malacological data made available by public agents. In our research a “case study” concerns geographical areas of low schistosomiasis endemicity and we did not have used neither confidential or human individual data nor animal tissue data. The use of the term 'case study' refers to situated investigations of processes and phenomena in a specific area or region. It should not be confused with 'disease cases' or those involving confidential human subjects data and information.

Considering this and the editorial guidelines on this issue we have understood that in the case of this study the *Ethical Statement* does not apply.

Results

Reviewer #1:

- Does the analysis presented match the analysis plan? Yes
- Are the results clearly and completely presented? Yes
- Are the figures (Tables, Images) of sufficient quality for clarity? Yes

Thanks for the positive feedback.

Thank you for your positive feedback.

Reviewer #2:
In summary, the results section of the manuscript seems to be well-executed, with a clear and thorough presentation of findings that match the pre-established analysis plan. The approach to evaluating and discussing the results is systematic and provides a solid foundation for the study's conclusions.

We appreciate your positive comments on our manuscript.

Reviewer #3: The analysis are excellent, results clearly presented with all the figures and tables being of high quality

Your positive feedback is appreciated.

Conclusions

Reviewer #1:

- Are the conclusions supported by the data presented? Yes
- Are the limitations of analysis clearly described? Yes
- Do the authors discuss how these data can be helpful to advance our understanding of the topic under study? Yes
- Is public health relevance addressed? Yes

We appreciate your positive feedback.

Reviewer #2:

The conclusion section effectively summarizes the study's findings, is supported by the data, and acknowledges its limitations. The authors also successfully link their research to its potential impact on public health policy and practice, particularly in the context of disease surveillance and control. This approach not only strengthens the study's academic contribution but also its practical utility for public health initiatives.

We appreciate your positive comments on our manuscript.

Reviewer #3:

The study conclusions are fully and adequately supported by the data presented
The study limitations are clearly described
The public health relevance is addressed
The authors have discussed conclusively the importance of their data to the advancement of the topic under study

Thank you for highlighting these positive aspects.

Editorial and Data Presentation Modifications

Reviewer #1:

My comments are shown in the text of the attached MS.

Lines 16, 449, 645, 654, 655, Fig 4 and Abstract: The suggestions were accepted and the text was updated.
Lines 98-108: The text was removed from Introduction as suggested.
Italics was used for the name of the species in the References.
Lines 138-140: According to the findings of Palasio et al. (2019), every *Biomphalaria* sample gathered in the field tested negative for *S. mansoni* cercariae in laboratory.

Reviewer #2:

1. Lines 142-167: The article utilizes multiple data sources, including remote sensing imagery, census data, and socio-economic data. This is a strength of the study as it allows for a multifaceted analysis of the issue. However, is it possible that differences in data quality and resolution could affect the accuracy of the results?

We would like to thank reviewer #2 for bringing up this point and allowing us to clarify it further. The methodology adopted works at the landscape scale and brings a mosaic of cells, where each cell of [500x500] meters represents a landscape unit. Therefore, the set of these cells covers the entire study area. From a technical point of view, these cells make up a new spatial support where data from different sources will be harmonized and integrated through operations that fill in these cells using specific operators (spatial, statistical, and others). Each different database (heterogeneous data sources) has its data treated in an appropriate manner, as described in the methodology section. This technique is referred to in operations on spatial data as the geometric support change operation. Data from different sources and different geometries are integrated into a new common spatial (geometric) support, in this case a grid of regular cells. This technique is the most widely used in order to overcome the challenge of integrating data from different formats, scales, resolutions and delimitations into the same spatial unit of analysis. An important factor in using this support change technique is deciding on the size of the cell. In the case of our study we tested several cell sizes ([100x100], [250x250], [500x500] and [1000x1000]). We evaluated the behavior of the cell filling operators when using the different sizes. Considering the limitations imposed by real data, the [500x500] cell size proved to be more suitable for accommodating the information extracted from the different sources used. This made it possible to obtain a more disaggregated form of distribution of the multiple data sources with different resolutions. However, the accuracy of the results can always be improved, as long as the databases are also improved. Sensitive tests can be applied in relation to cell size. In the work we are developing, we will expand this methodology to the entire Paranapanema Basin region. We will carry out a sensitivity assessment of the model's response to different cell sizes, which at the moment has been in a preliminary phase needing some adjustment.

2. Lines 334-341: The use of Boosted Decision Tree as a classification method in the study is a reasonable choice because it can handle complex nonlinear relationships. It may be worth considering providing additional details on the rationale for model selection and the parameter tuning process to enhance transparency in the methods section.

Thank reviewer #2 for your suggestion. We have updated the manuscript to include more details:

Lines 311-315: "Boosting, which is based on an adaptive learning method, aims to improve predictive ability by reducing both bias and variance in the model [48, 49]. This method can capture complex non-linear relationships, patterns and interactions among features, handling different types of data, including numerical and categorical variables."

Lines 318-324: "Being a supervised method, the decision tree classification requires the use

of samples provided by the analyst to train the classifier and assess quality of the classification. The boosting procedure used all the variables present in Table 2 and generated the maximum number of 300 decision trees. The errors of the individual trees during training ranged from 6.9% to 39.0%. The use of boosting, by the voting system, minimizes these tree individual errors by combining the 300 decision tree results trying to reach an error as close to zero as possible."

3. Has the study considered the generalizability of the model to other regions or under different conditions? Validating the applicability of the model in new areas is important.

The condition of small-scale agriculture near urban areas with a history of schistosomiasis cases among workers is a specific reality of the study area. The typology created for landscape classification respected these local characteristics of schistosomiasis transmission. However, the study utilized nationwide data coverage to facilitate the replication of the methodology in other areas that would require some adaptations in relation to the characteristic of the regional landscape pattern, creating a specific typology to reflect local dynamics. Essentially, using a new typology suited to the new landscape being analyzed, the methodology remains the same. In this sense, the method can be used and applied for different landscapes. For regions where the typology remains the same or is very similar, the methodology works and therefore, in a way, the model is generalizable.

4. Lines 608-610: The conclusion mentions the potential impact of climate change on the spread of schistosomiasis. How do these changes specifically affect risk maps, and what are the possible adaptation strategies?

Climate change affects the risk map by requiring frequent updates to encompass changes in drainage patterns expected with increased frequency and volume of rainfall. If this increased drainage is not accompanied by WASH (Water, Sanitation, and Hygiene) strategies, the potentially active areas could expand. An adaptation strategy for the map would involve integrating predictive drainage models into the data used for landscape classification.

5**.** Lines 612-613: The study provides detailed risk maps, which are highly useful for disease control and intervention strategies. Have considerations been made on how these maps specifically impact policy-making and fieldwork?

Thank reviewer #2 for your observation. We have updated the manuscript to answer this question:
Lines 503-507: In the context of low endemicity, the map emerges as an important tool to support localized action strategies in hot spots to eliminate schistosomiasis and avoid its resurgence. Additionally, the map can also guide public efforts to expand coverage of basic sanitation in areas requiring such investments, specifically in potentially active landscapes for schistosomiasis.

6. Lines 622-626: The transmission of schistosomiasis is a dynamic process influenced by

multiple factors. Has there been consideration for regularly updating risk maps to reflect changes in environmental and socio-economic conditions?

Yes, it has been considered to update the map for the study area when new data become available. New data from the Brazilian demographic census (2020-2022 census) will soon be available and socio-economic data can be updated, for example. There is also a consideration to generate a more refined scale map for dense urban areas using data generated from high-resolution satellite images.

Reviewer #3:
Author summary- Line 3, put citation in to brackets (Sambon , 1907)

The change has been made.

Methodology: study area: Line 138- repetition of the word 'from'

The change has been made.

Methodology: Physical landscape data: Lines 173-175: Was potential agricultural areas which could be high potential areas for human parasite interactions captured under Open Street Map (OSM)?

OpenStreetMap (OSM) provides a land use and land cover layer that includes agriculture class. However, due to its collaborative mapping initiative and the lack of systematic data production and accuracy assessment, there is insufficient data completeness in the study area. OSM data covers only 3.4% of the Médio Paranapanema water basin with land use and land cover classification.

Include an ethical statement for the study-Did the study obtain ethical approval?

The study was not submitted for ethical approval as it did not have used either confidential or human individual data nor animal tissue data.

Summary and General Comments

Reviewer #1:
The theme of the manuscript is undoubtedly of great interest to public health as schistosomiasis is currently among the most important neglected diseases in the tropics. In the specific case of Brazil, the focus of this Manuscript, although the last prevalence survey carried out indicated a significant decrease in the prevalence of schistosomiasis, there are still areas with high prevalence, in addition to the persistence of those with low prevalence and some focal areas of schistosomiasis mansoni. In recent years, there is a consensus that the

control and possible elimination of schistosomiasis requires the application of several integrated measures, as it is well presented in this Manuscript. The authors present here a well-structured strategy, involving several relevant aspects in the epidemiology of schistosomiasis transmission (orbital sensing remote images, environmental, socioeconomic, epidemiological and malacological data) aiming to define and
characterize the scenario of different landscapes and their association with potential areas of transmission of schistosomiasis, in a region traditionally recognized as having low endemicity in the Paranapanema River Basin, state of São Paulo. Based at a landscape scale the authors present here a map of schistosomiasis risk transmission across the Paranapanema basin, which undoubtedly constitutes an useful tool for schistosomiasis surveillance and control programs.

The methodology is appropriate. The results obtained will certainly contribute to the control of schistosomiasis in the study area, in addition to being able to be replicated in other endemic regions.

Therefore, I recommend the publication of the manuscript. Some comments were made throughout the attached Ms., aiming to clarify some points for the reader and I suggest that they be considered.

We appreciate your positive comments on our manuscript.

Reviewer #2:
This article presents a comprehensive and in-depth study that successfully mapped the risk landscapes for schistosomiasis in the Middle Paranapanema river basin of Brazil by integrating a variety of data sources and employing advanced machine learning techniques. The research team identified distinct landscape types associated with the transmission potential of schistosomiasis through meticulous Geographic Information Systems (GIS) analysis and landscape ecology methods, which are crucial for formulating disease control and elimination strategies.

The methodology section of the article is detailed, describing the data collection and analysis processes, including the classification of land use and land cover, utilization of road databases, and interpretation of radar imagery. These elements provide a solid technical foundation for the study. Furthermore, the use of a Boosted Decision Tree for supervised classification to generate landscape risk maps with high accuracy demonstrates the reliability and effectiveness of the approach.

We appreciate your positive comments on our manuscript.

Reviewer #3:
This is quite a comprehensively well written manuscript that has employed integrated landscape-based approaches (remote sensing, environmental, socioeconomic,

epidemiological, and malacological data) to aid surveillance and control strategies for schistosomiasis in low-endemic areas. The authors have clearly brought about an innovative methodology that can be applied in schistosomiasis surveillances especially in areas that are translating from control to elimination of schistosomiasis.

We appreciate your positive comments on our manuscript.
