# Supplementary material for: Mapping schistosomiasis risk landscapes and implications for disease control: A case study for low endemic areas in the Middle Paranapanema river basin, São Paulo, Brazil
Source: PLoS Negl Trop Dis. 2024 Nov 4;18(11):e0012582. doi: 10.1371/journal.pntd.0012582 (PMC11563476; doi:10.1371/journal.pntd.0012582)
Supplement: Attachment — Submitted filename: renamed_729f4.docx [file pntd.0012582.s008.docx]

This article presents a comprehensive and in-depth study that successfully mapped the risk landscapes for schistosomiasis in the Middle Paranapanema river basin of Brazil by integrating a variety of data sources and employing advanced machine learning techniques. The research team identified distinct landscape types associated with the transmission potential of schistosomiasis through meticulous Geographic Information Systems (GIS) analysis and landscape ecology methods, which are crucial for formulating disease control and elimination strategies.

The methodology section of the article is detailed, describing the data collection and analysis processes, including the classification of land use and land cover, utilization of road databases, and interpretation of radar imagery. These elements provide a solid technical foundation for the study. Furthermore, the use of a Boosted Decision Tree for supervised classification to generate landscape risk maps with high accuracy demonstrates the reliability and effectiveness of the approach.

1. Lines 142-167: The article utilizes multiple data sources, including remote sensing imagery, census data, and socio-economic data. This is a strength of the study as it allows for a multifaceted analysis of the issue. However, is it possible that differences in data quality and resolution could affect the accuracy of the results?

2. Lines 334-341: The use of Boosted Decision Tree as a classification method in the study is a reasonable choice because it can handle complex nonlinear relationships. It may be worth considering providing additional details on the rationale for model selection and the parameter tuning process to enhance transparency in the methods section.

3. Has the study considered the generalizability of the model to other regions or under different conditions? Validating the applicability of the model in new areas is important.

4. Lines 612-613: The study provides detailed risk maps, which are highly useful for disease control and intervention strategies. Have considerations been made on how these maps specifically impact policy-making and fieldwork?

5. Lines 622-626: The transmission of schistosomiasis is a dynamic process influenced by multiple factors. Has there been consideration for regularly updating risk maps to reflect changes in environmental and socio-economic conditions?

6. Lines 608-610: The conclusion mentions the potential impact of climate change on the spread of schistosomiasis. How do these changes specifically affect risk maps, and what are the possible adaptation strategies?
